# Fractional laser exposure induces neutrophil infiltration (N1 phenotype) into the tumor and stimulates systemic anti-tumor immune response

**DOI:** 10.1371/journal.pone.0184852

**Published:** 2017-09-18

**Authors:** Masayoshi Kawakubo, Shadmehr Demehri, Dieter Manstein

**Affiliations:** 1 Cutaneous Biology Research Center, Department of Dermatology, Massachusetts General Hospital Research Institute, Charlestown, MA, United States of America; 2 Harvard Medical School, Boston, MA, United States of America; Istituto Superiore di Sanità, ITALY

## Abstract

**Background:**

Ablative fractional photothermolysis (aFP) using a CO_2_ laser generates multiple small diameter tissue lesions within the irradiation field. aFP is commonly used for a wide variety of dermatological indications, including treatment of photodamaged skin and dyschromia, drug delivery and modification of scars due to acne, surgical procedures and burns. In this study we explore the utility of aFP for treating oncological indications, including induction of local tumor regression and inducing anti-tumor immunity, which is in marked contrast to current indications of aFP.

**Methodology/Principal findings:**

We used a fractional CO_2_ laser to treat a tumor established by BALB/c colon carcinoma cell line (CT26.CL25), which expressed a tumor antigen, beta-galactosidase (beta-gal). aFP treated tumors grew significantly slower as compared to untreated controls. Complete remission after a single aFP treatment was observed in 47% of the mice. All survival mice from the tumor inoculation rejected re-inoculation of the CT26.CL25 colon carcinoma cells and moreover 80% of the survival mice rejected CT26 wild type colon carcinoma cells, which are parental cells of CT26.CL25 cells. Histologic section of the FP-treated tumors showed infiltrating neutrophil in the tumor early after aFP treatment. Flow cytometric analysis of tumor-infiltrating lymphocytes showed aFP treatment abrogated the increase in regulatory T lymphocyte (Treg), which suppresses anti-tumor immunity and elicited the expansion of epitope-specific CD8^+^ T lymphocytes, which were required to mediate the tumor-suppressing effect of aFP.

**Conclusion:**

We have demonstrated that aFP is able to induce a systemic anti-tumor adaptive immunity preventing tumor recurrence in a murine colon carcinoma in a mouse model. This study demonstrates a potential role of aFP treatments in oncology and further studies should be performed.

## Introduction

Fractional Photothermolysis (FP) is a laser-assisted treatment producing a pattern of microscopic treatment zones (MTZs) in biological tissue [1]. Ablative FP (aFP) generates MTZs which are characterized by a central “hole” of physically-removed (ablated) tissue, typically surrounded by a small cuff of thermally damaged tissue, while non-ablative FP (nFP) generates MTZs by small zones of thermally damaged tissue in the absence of any centrally ablated (removed) tissue. [2, 3]. The diameter of the MTZs is typically less than approximately 0.5 mm. FP techniques are characterized by direct exposure of only a small fraction of the tissue to the laser radiation (typically only 5–27% of the treatment area), with most of the tissue being spared or unexposed [4]. FP is currently used for a wide spectrum of dermatological indications including, but not limited to, treatment of photodamaged skin, dyschromia, rhytides, and various kind of scars including acne, surgical and burn scars [1, 5–8]. Ablative FP has been used in combination with photodynamic therapy (PDT) to treat skin cancer; however, in conjunction with this indication, aFP is mainly used to provide enhanced topical delivery of the photosensitizing drug by increasing permeability of the skin [9, 10]. Non-ablative FP has been used to treat precancerous skin lesions (actinic keratoses). However such treatments have been limited to direct irradiation of local skin regions, and no studies to date have investigated production of systemic effects using FP methods [11]. Also FP is typically not used to treat tumors as the current paradigm of tumor treatment is to destroy the entire tumor volume rather than inducing damage only to a small portion of the tumor.

Photodynamic therapy (PDT) has been used successfully for local cancer therapy. Various types of cancer have been treated with PDT including, but not limited to, skin cancer [10]], lung cancer [12, 13], bile duct cancer [14, 15], and pancreatic cancer [16]. The response to PDT treatment is dependent on the cancer type and cell lines present. Mroz et al. [17] reported in a murine cancer model that PDT of an intradermally inoculated CT26.CL25 tumor induced local remission as well as a systemic tumor-specific immune response, resulting in regression of a remote, untreated tumor (abscopal effect). It has thus been observed that PDT is able to induce a systemic, tumor specific anti-tumor immunity. However, PDT has some shortcomings because it is a drug-device combination treatment that requires the administration of the photosensitizing drug in a dose dependent and time-sensitive manner. The PDT effect also depends on the bioavailability of the photosensitizer and requires an oxygen rich environment [18–20]. Both requirements can be a challenge within tumors, which are often characterized by blood vessel compression and hypoxemia due to the tumor growth [21, 22]. As most non-dermatological tumors require systemic application of the photosensitizer, the resulting requirement for prolonged light avoidance of patients is another downside of systemically delivered PDT.

In contrast, FP is a laser-only treatment and does not require bioavailability of a drug within the tumor tissue. FP of skin is a simple and quickly performed procedure, which is routinely performed for the above-mentioned dermatological, non-cancerous indications. FP of non-dermatological tissues could also be performed easily, although anatomical site-specific laser delivery applicators might be required for non-dermal tissue irradiation. Motivated by these potential advantages as compared to PDT treatments, we have investigated effects of aFP itself in the absence of any photosensitizer on systemic tumor-specific immune responses. Our objective was to investigate if aFP is has some promise to induce any anti tumor effects at all which is in marked contrast to the current applications of FP.

## Results

### Fractional laser beam penetrates the skin and the tumor

**Fig 1:**
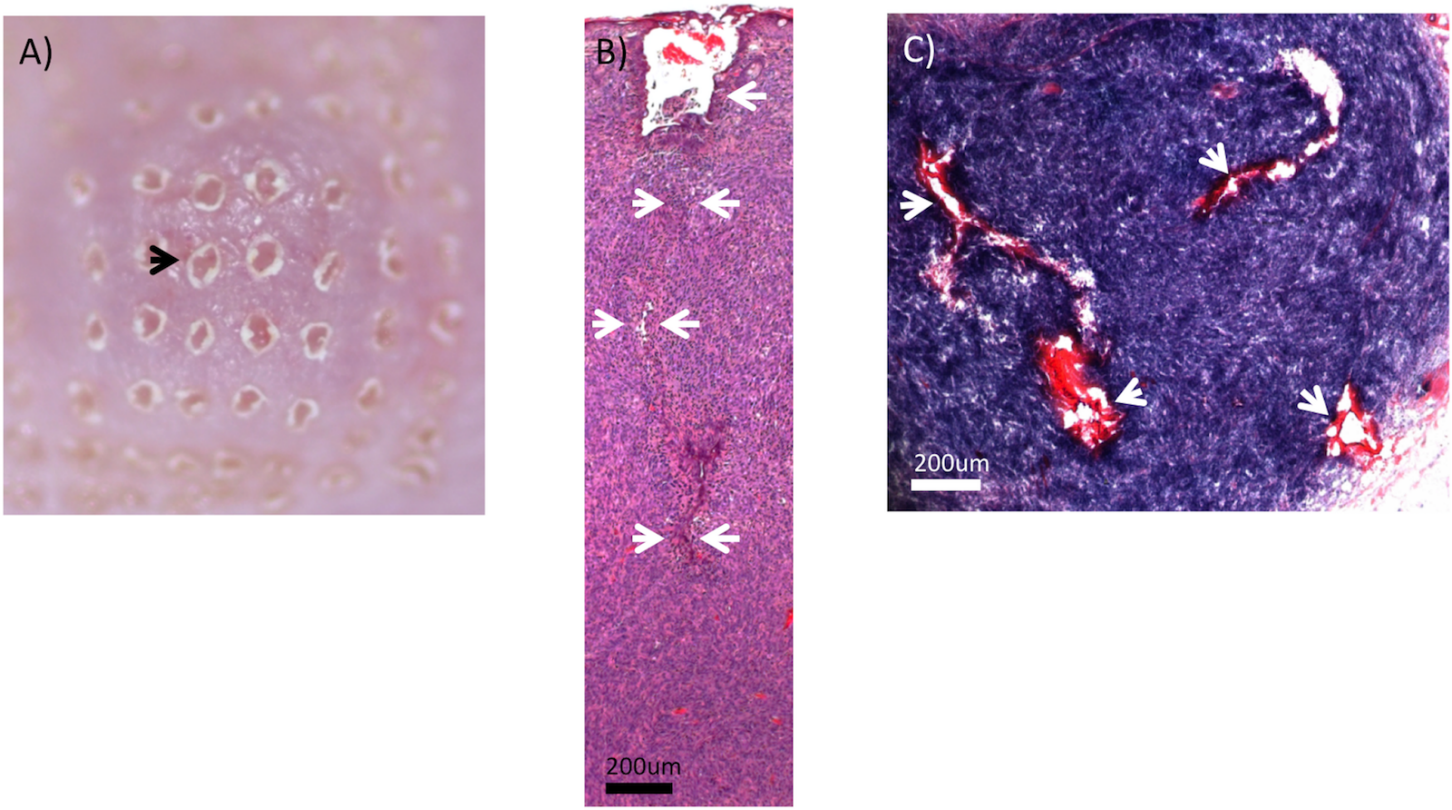
Ablative fractional photothermolysis. (A) photo of skin surface appearance immediately after ablative fractional photothermolysis (100 mJ pulse energy). Black arrow indicates one of the laser-generated holes formed by tissue vaporization/ablation. There is an absence of graying or blistering immediately after laser exposure, which also suggests an absence of major thermal injury. (B) H&E-stained CT26.CL25 tumor immediately after the ablative fractional photothermolysis (aFP) laser treatment with a pulse energy of 100mJ. White arrows indicate an ablated hole which is characteristic of aFP procedures. The ablated hole appeared to be collapsed and distorted within the tumor tissue. (C) CT26.CL25 tumor immediately after aFP, stained by NBTC staining that shows vital cells as a blue color. White arrows indicate dead cells caused by physical effects of the laser treatment. Most of the tissue within the aFP volumes exhibited a blue staining, indicating an absence of widespread thermal injury or tissue bulk heating.

Fig 1A shows the appearance of the skin surface immediately after ablative fractional laser exposure. The fairly regular alignments of the ablated holes can be seen. There is absence of graying or blistering of the skin immediately after laser exposure, which suggests an absence of thermal bulk injury. The CO_2_ laser-induced ablative MTZs reached well into the tumor tissue. We have analyzed multiple samples and found reproducible an average lesion depth of approximately 3.1 mm from the surface of the skin using a laser energy of 100 mJ (Fig 1B). The ablated holes, which are characteristic of aFP procedures, could be seen. However, these ablated holes appeared to be collapsed and distorted within the tumor tissue. Representative horizontal sections of the aFP-treated tissue were consistent with the morphology seen in the vertical sections (Fig 1C). The horizontal sections exhibited a regular pattern consistent with the scanning pattern of the laser exposures. The thermal injury zone, as marked by loss of blue NBTC stain, exhibited a thickness of less than 40 μm surrounding the laser induced holes in the tissue. Diameter of the MTZ (The thermal injury zone + laser-ablated holes) was approximately 250 μm. Assuming a cylinder geometry (volume = height x diameter^2^ x π/ 4) the total volume of single MTZ in the tumor was 0.15 mm^3^. This is an estimate as it is difficult to calculate the exact volume because the channels were partially collapsed (decreased diameter) but also exhibited some irregular lateral protrusions of the channels (increased thermally affected volume).

The projected MTZ / tumor surface ratio was approximately 8%. However MTZs did not reach to approximately more than 2/3 of the tumor depth. Therefore the total MTZ / tumor volume ratio was approximately 5%. It was notable that the lumen of the laser-ablated holes were irregular and exhibited some irregular lateral protrusions of damaged tissue that extended into some of the spaces between the irradiation locations of the scanning pattern. Such protrusions would be consistent with hot steam jets originating from the individual laser-irradiated sites due to the explosive nature of the laser-assisted ablation process. Most of the tissue within the aFP treatment volumes exhibited a dark blue NBTC staining, indicating an absence of widespread thermal injury or tissue bulk heating.

### aFP is significantly effective against local primary tumors

**Fig 2:**
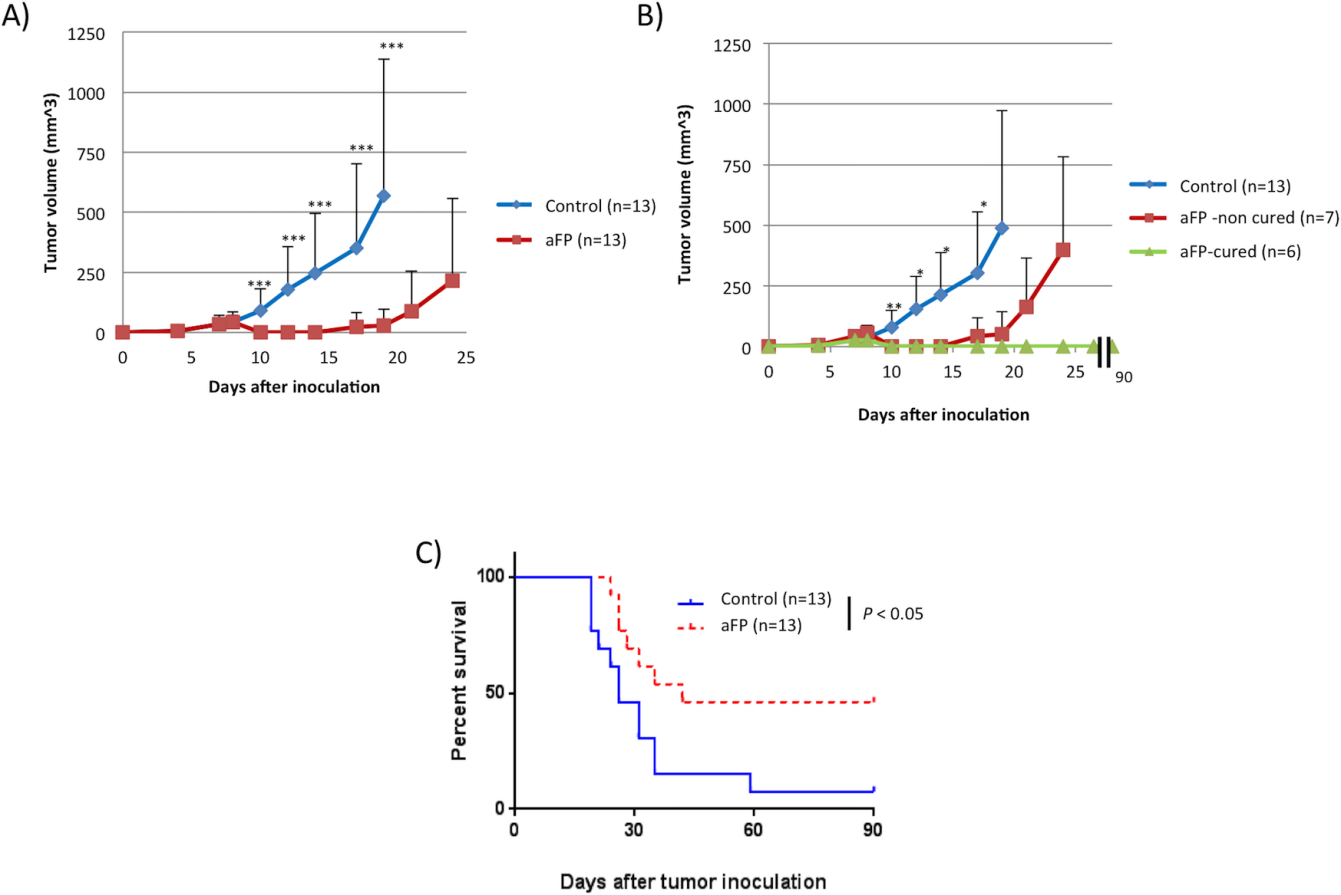
Tumor volume and survival curves after aFP treatment. (A) tumor volume curves of mice in the control group and aFP group after tumor inoculation. *** *P* < 0.0001. The bars represent SD. (B) tumor volume curve of mice in the control group, and tumor volume curves of cured mice and non-cured mice which are split from original curve in aFP group. * *P* < 0.01, ** *P* < 0.005 comparing control to aFP-non cured group. The bars represent SD. (C) Kaplan-Meier survival curves of mice receiving tumor inoculation. The significance values for the difference between the survival curves are: control vs. FP (*p* < 0.05).

aFP laser irradiation was performed 8 days after tumor inoculation. The aFP significantly led to a reduction in tumor volume and growth rate of beta-gal antigen positive CT26.CL25 tumors after the treatment (*P* < 0.005: Fig 2A). Significant tumor reduction was observed even in non-cured CT26.CL25 tumor-bearing mice (*P* < 0.005: Fig 2B). 7 of 13 mice (53%) with CT26.CL25 tumor treated with aFP were euthanized between days 24 and 42 due to local tumor recurrence and regrowth (Fig 2C). Tumors shrank in 6 mice of the aFP group, but such shrinkage occurred in only 1 mouse from the control group (Fig 2C). The significance value for the difference between the survival curves is: control vs. aFP (p<0.05).

Repeated experiments are shown in S2 Fig.

### aFP induces development of long term anti-tumor immunity

**Fig 3:**
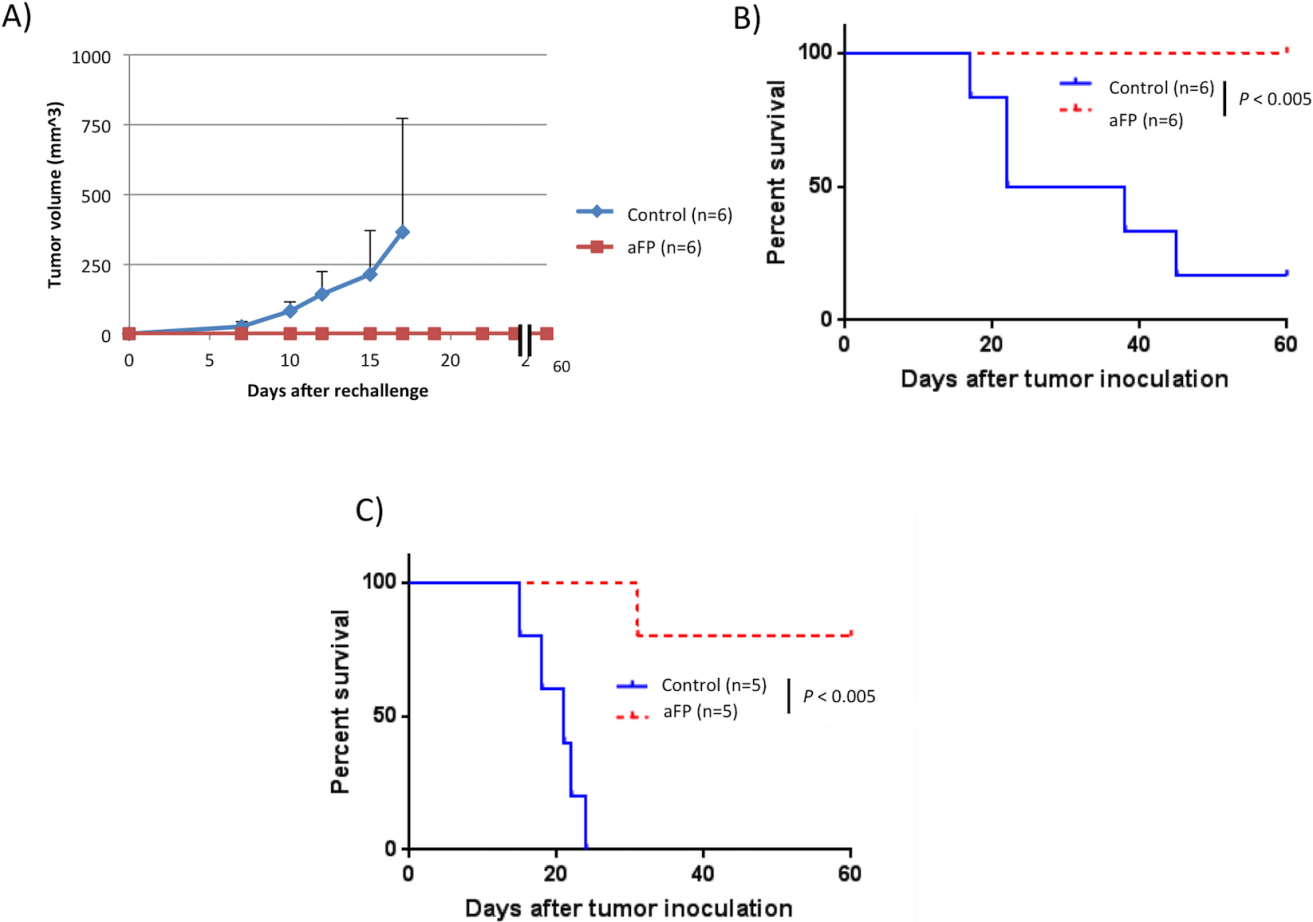
Tumor volume and survival curves after rechallenge test. (A) mean tumor volume curves of the mice in the control group and survival mice after the rechallenge test. These mice were each inoculated with 3.5 x 10^5 CT26.CL25 cells intradermally in the contralateral (right) thigh against previous inoculation. Tumors on the survival mice in the aFP group did not appear to progress. (B) Kaplan-Meier survival curves of mice receiving the rechallenge test with CT26.CL25 cells. The significance values for the difference between the survival curves are: control mice vs. survival mice (p<0.005). (C) Kaplan-Meier survival curves of mice receiving the rechallenge test with CT26 wildtype cells which is parental tumor of CT26.CL25 cells. The significance values for the difference between the survival curves are: control mice vs. survival mice (p<0.005).

To assess the presence of any induced systemic immunity, we performed a rechallenge experiment following the aFP laser treatment. Six mice in the aFP group which survived for more than 90 days after the aFP, were subsequently inoculated intradermally with CT26.CL25 in the contralateral (right) thigh. Six age-matched naive mice were inoculated with the same number of CT26.CL25 cells, respectively, in the right thigh as a control. The tumor on the naive mice in the control groups progressed over time (Fig 3A). Tumors on the survival mice in the aFP-treated group did not appear to progress (Fig 3A). They remained tumor–free for at least another 60 days following the inoculation (Fig 3B). The significance values for the differences between the survival curves are: control mice vs. survival mice (p<0.005).

To investigate the role of artificial tumor antigen (beta-gal) in developing anti tumor immunity, we performed a rechallenge experiment using CT26 wild type (CT26WT) colon carcinoma cells, which are beta-gal negative parental carcinoma of CT26.CL25 cells. Mice cured from CT26.CL25 tumor and survived tumor free for 90 days after aFP treatment were inoculated with CT26WT cells. Interestingly 4 of 5 survival mice rejected CT26WT tumor and remained tumor–free for another 60 days following the inoculation (Fig 3C).

Repeated experiments are shown in S3 Fig.

### aFP induce accumulation of the N1 neutrophils

**Fig 4:**
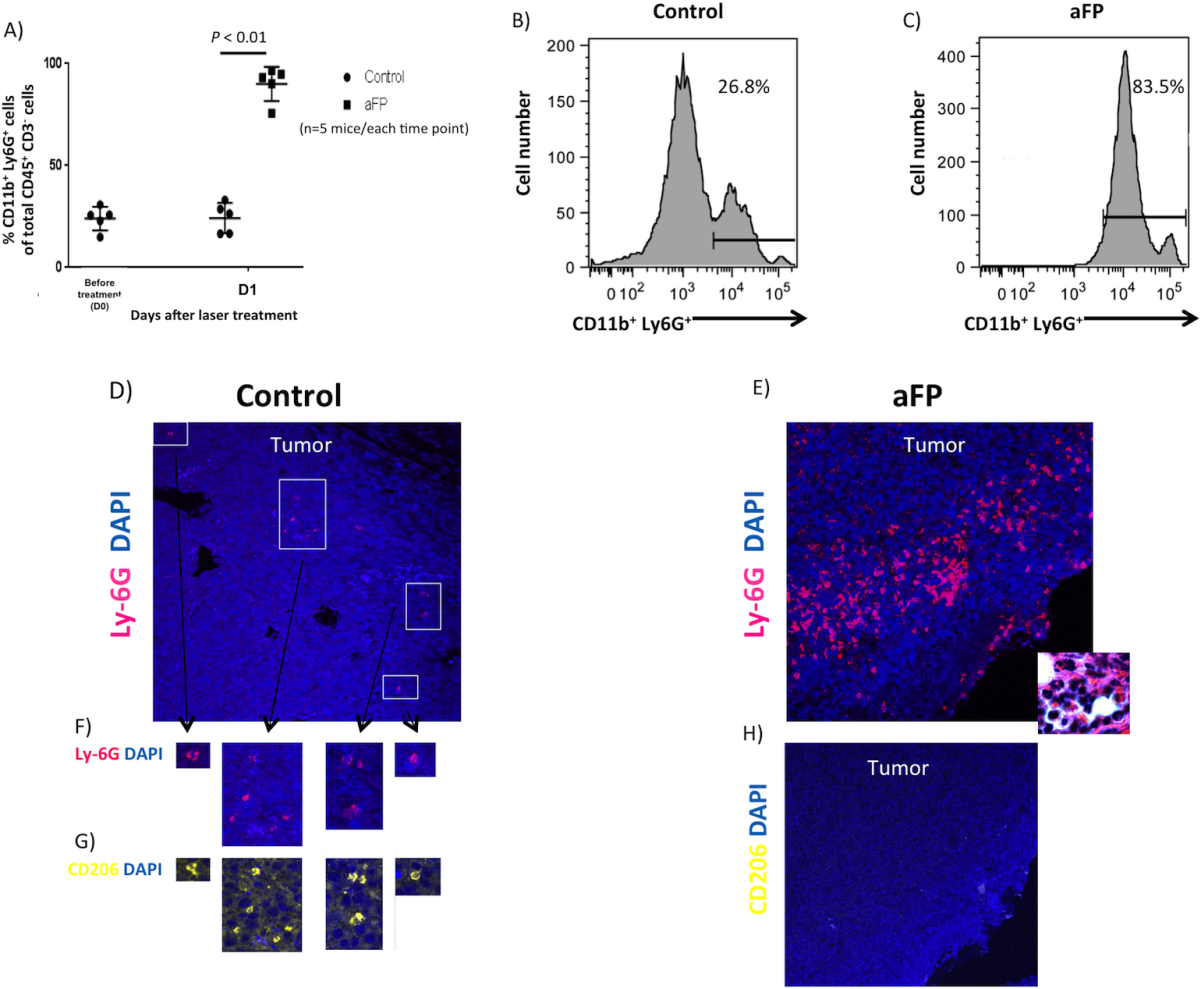
Immunochemical staining and flow cytometric analysis for tumor infiltrating neutrophils. (A) proportion of neutrophil compared with CD45^+^CD3^-^ leukocytes in the tumor of flow cytometric analysis. (B and C) representative images of flow cytometry for neutrophil on day 1 after aFP in the control group and aFP group respectively. The number in the figures represents proportion of neutrophil compared with CD45^+^CD3^-^ leukocytes. (D and E) immunohistochemical staining for neutrophil in the tumor 1 days after aFP in the control group and aFP group respectively. Cells stained as red color are neutrophils. Inset shows multi nucleated neutrophils as the dominant immune infiltrate seen in H&E-stained aFP-treated tumor. (F) magnified image of neutrophil in figure (4D, G and H) immunohistochemical staining for CD206-expressing neutrophil in the tumor 1 days after aFP in the control group and aFP group respectively. Cells stained as yellow color are neutrophils expressing CD206.

**Fig 5:**
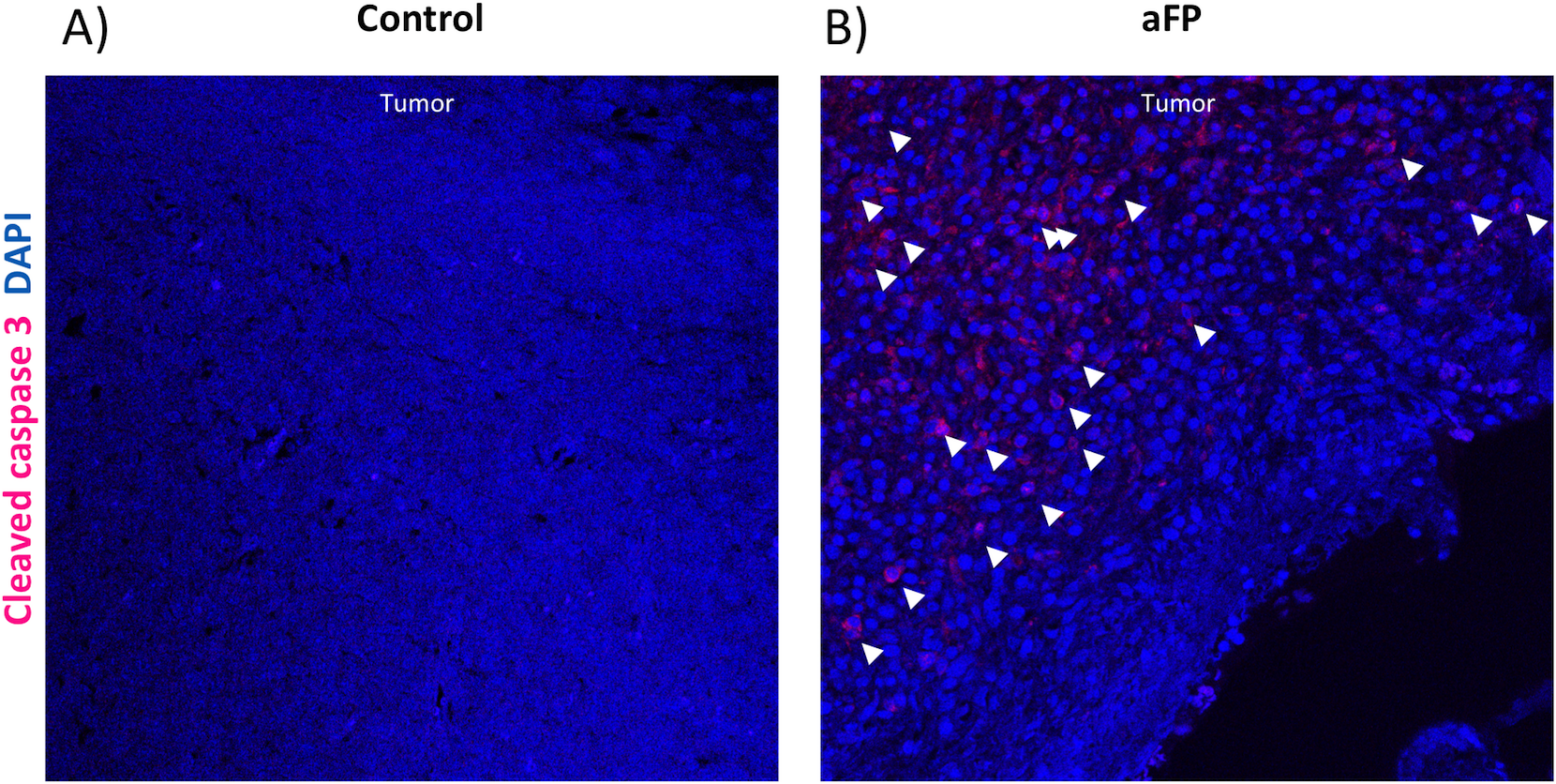
Immunohistochemical staining for apoptotic tumor cells. (A and B) Immunohistochemical staining for apoptotic cells in the tumor 1 day after aFP in the control group and aFP group respectively. Representative images are shown. Cells stained as red color, which are indicated by white arrow heads are apoptotic cells.

To investigate whether innate immunity was induced after the fractional laser treatment, flow cytometry and immunohistochemistry for neutrophil was performed. Flow cytometric analysis showed aFP led to a significant increase in neutrophil on day 1 after aFP, proportions of neutrophil compared with CD45^+^ CD3^-^ leukocytes were significantly higher in the aFP group than in control (*p* < 0.01: Fig 4A). Fig 4B and 4C are representative images of flow cytometry on day 1 after aFP in the control group and aFP group respectively. The number in the figures represents proportion of neutrophils compared with CD45^+^ CD3^-^ leukocytes. Although tumor volume in the aFP group were smaller than in control group, more neutrophils could be counted (Fig 4B and 4C). We also detected NK cells, B lymphocytes and macrophage using flow cytometry however there were no significant differences between both groups (S1A Fig). Immunohistochemical staining also showed that the neutrophils apparently accumulated in the tumors in the aFP group more than in the control group on day 1 after aFP (Fig 4D–4F). Tumor-infiltrating neutrophils in control group showed CD206 expression, which indicates N2 protumorigenic neutrophil [23] (Fig 4G). However the neutrophil in aFP group did not show CD206 expression, which indicates N1 antitumorigenic neutrophil [23] (Fig 4H). These results imply aFP induces infiltration of antitumorigenic neutrophil into the tumor. Additionally, we found there were apoptotic tumor cells around the site of N1 neutrophils accumulation within the tumor in the aFP group (Fig 5A and 5B).

Repeated experiments are shown in S4–S6 Figs.

### aFP abrogates the increase in regulatory T lymphocyte (Treg) and develop epitope specific CD8^+^ T lymphocytes

**Fig 6:**
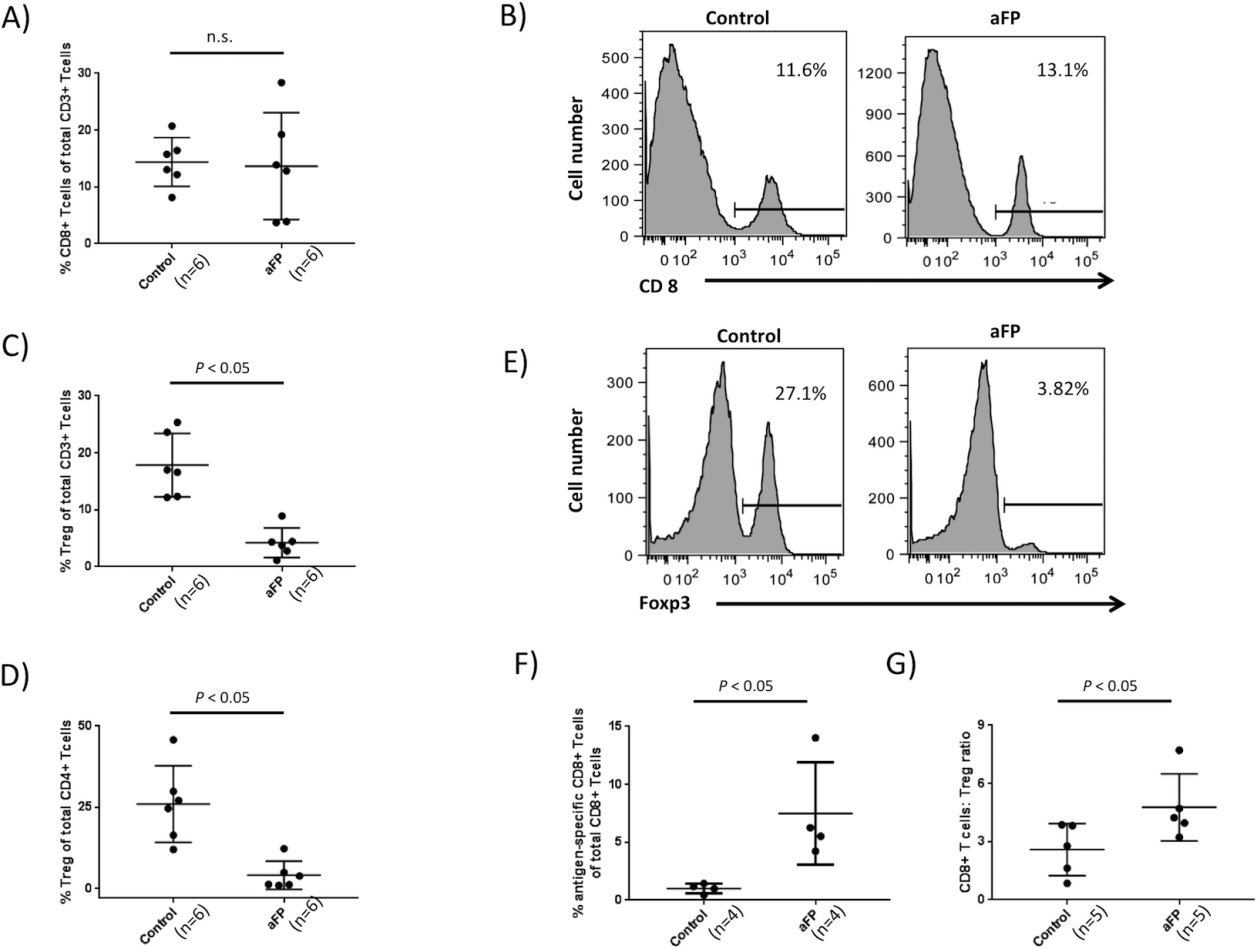
Flow cytometric analysis for tumor infiltrating lymphocytes. (A) proportion of CD8^+^ T lymphocytes compared with CD3^+^ T lymphocytes in the tumor. (B) representative images of flow cytometry for CD8^+^ T lymphocytes on day 5 after aFP in the control group and aFP group respectively. The number in the figures represents proportion of CD8^+^ T lymphocytes compared with CD3^+^ T lymphocytes. (C and D) proportion of Treg compared with CD3 and CD4^+^ T lymphocytes in the tumor respectively. (E) representative images of flow cytometry for Treg on day 5 after aFP in the control group and aFP group respectively. The number in the figures represents proportion of CD4^+^ Foxp3^+^ Treg compared with CD4^+^ T lymphocytes. (F) proportion of beta-gal epitope specific CD8^+^ T lymphocytes compared with total CD8^+^ T lymphocytes in the tumor. (G) proportion of CD8^+^ T lymphocytes compared with Treg in the tumor.

To investigate if the adaptive immune system was affected by aFP, we measured the ratio of Treg and CD8^+^ T lymphocytes in tumor infiltrating lymphocytes (TILs) using flow cytometry. Each time point included 6 mice. We found that there was no significant difference between the groups in a proportion of CD8^+^ T lymphocytescompared with CD3^+^ T lymphocytes(Fig 6A). Fig 6B are representative images of flow cytometry on day 5 after aFP in the control group and aFP group respectively. The number in the figures represents proportion of CD8^+^ T lymphocytes compared with CD3^+^ Tlymphocytes. However aFP led to a significant decrease in Treg which suppresses the function of the immunocell on day 5 after aFP, proportions of Treg compared with CD3 and CD4^+^ T lymphocyteswere significantly lower in the aFP group than in control (*p* < 0.05: Fig 6C and 6D). Fig 6E are representative images of flow cytometry on day 5 after aFP in the control group and aFP group respectively. The number in the figures represents proportion of CD4^+^ Foxp3^+^ Treg compared with CD4 positive T lymphocytes. This observation indicates that immunosuppression by Treg in the aFP group is less than in the control group. Additionally we investigated whether beta-gal epitope (TPHPARIGL) specific CD8^+^ T lymphocytes were elicited or not. To detect them, beta-gal specific pentamer was incubated with TILs, anti-CD8, CD4 and Foxp3 antibodies. We found that aFP led to a significant increase in beta-gal specific CD8^+^ T lymphocytes compared with total CD8^+^ T lymphocytes on day 5 after aFP (*p* < 0.05: Fig 6F), and moreover in beta-gal epitope specific CD8^+^ T lymphocytes compared with Treg (*p* < 0.05: Fig 6G).

Repeated experiments are shown in S7 Fig.

### aFP induces and Treg suppresses the cytotoxic function of CD8^+^ T lymphocytes

**Fig 7:**
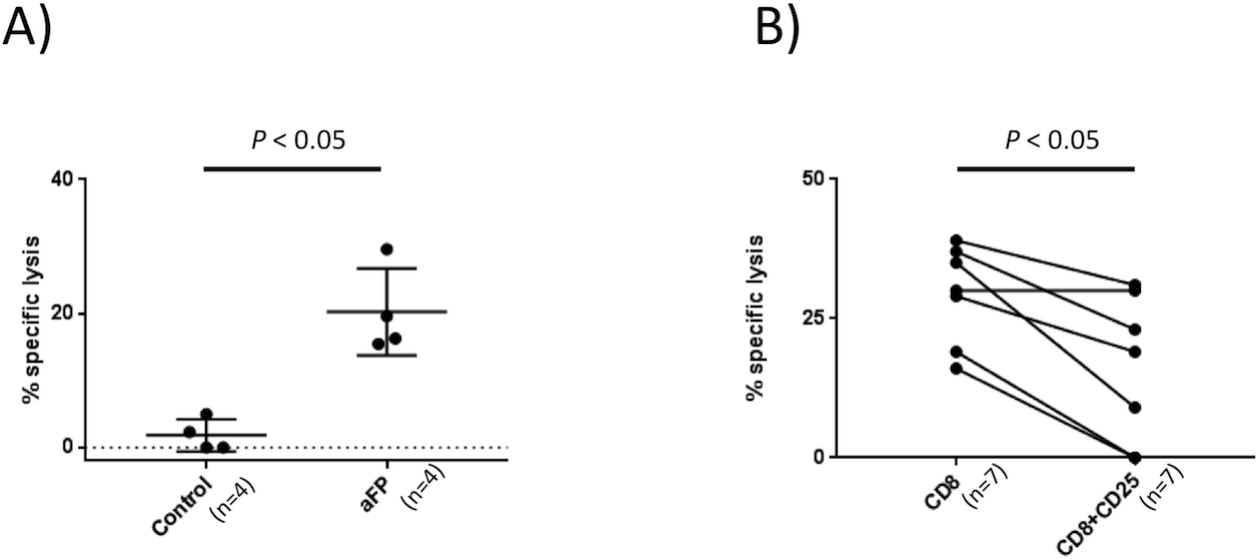
Cytotoxicity assay and Treg function examination. (A) the number in the figures represents % specific lysis of sorted CD8^+^ T lymphocytes from TILs against CT26.CL25 cells. Average specific lysis against CT26.CL25 cells in the aFP group was significantly higher than in the control group (*P* < 0.05). (B) percentage of specific lysis CT26.CL25 by sorted CD8^+^ T lymphocytes from TILs with and without CD4^+^CD25^+^ T lymphocytes (sorted from tumor drainage lymph node).

To investigate the involvement of adaptive immunity in aFT effect against cancer, we measured CD8^+^ T lymphocytes-cytotoxicity against CT26.CL25 colon carcinoma cells using CD8^+^ T lymphocytes sorted from TILs 5 days after aFP treatment. Control mice had no treatment. As shown Fig 7A, cytotoxicity assay shows sorted CD8^+^ T lymphocytes from aFP-treated mice displayed significantly specific lysis comparing with control mice (*p* < 0.05; Fig 7A).

Moreover to investigate whether aFP-induced CD8^+^ T lymphocytes-cytotoxicity was suppressed by Treg, we measured the cytotoxicity with/without CD25^+^ (which is one of the marker of the Treg) positive CD4^+^ T lymphocytes after aFP treatment. Before the investigation, we measured the percentage of Foxp3^+^ T lymphocytes of total CD4^+^ CD25^+^ T lymphocytes was included in TIL and the drainage lymph node on day 13 (5 days after aFP treatment). We found that 52% in control group and 3% in aFP group of the total CD4^+^ CD25^+^ T lymphocytes in TIL were Foxp3 positive (S1B Fig). However 97% in control group and 95% in aFP group of the total CD4^+^ CD25^+^ T lymphocytes in the drainage lymph node were Foxp3 positive (S1C Fig). Therefore, we focused our study on CD4^+^ CD25^+^ T lymphocytes in the drainage lymph node as Treg as suppressor cells due to the high percentage of Foxp3^+^ cells of CD4^+^ CD25^+^ lymphocytes.

We found sorted CD8^+^ T lymphocytes co-cultured with CD4^+^ CD25^+^ T lymphocytes displayed significantly decrease of specific lysis against CT26.CL25 cells compared with co-cultured without CD4^+^ CD25^+^ T lymphocytes (*p* < 0.05; Fig 7B). These results indicate that Treg suppress the cytotoxic function of CD8^+^ T lymphocyte against cancer cells.

Repeated experiments are shown in S8 Fig.

### Adaptive immune system is necessary to eradicate cancer cell after aFP

**Fig 8:**
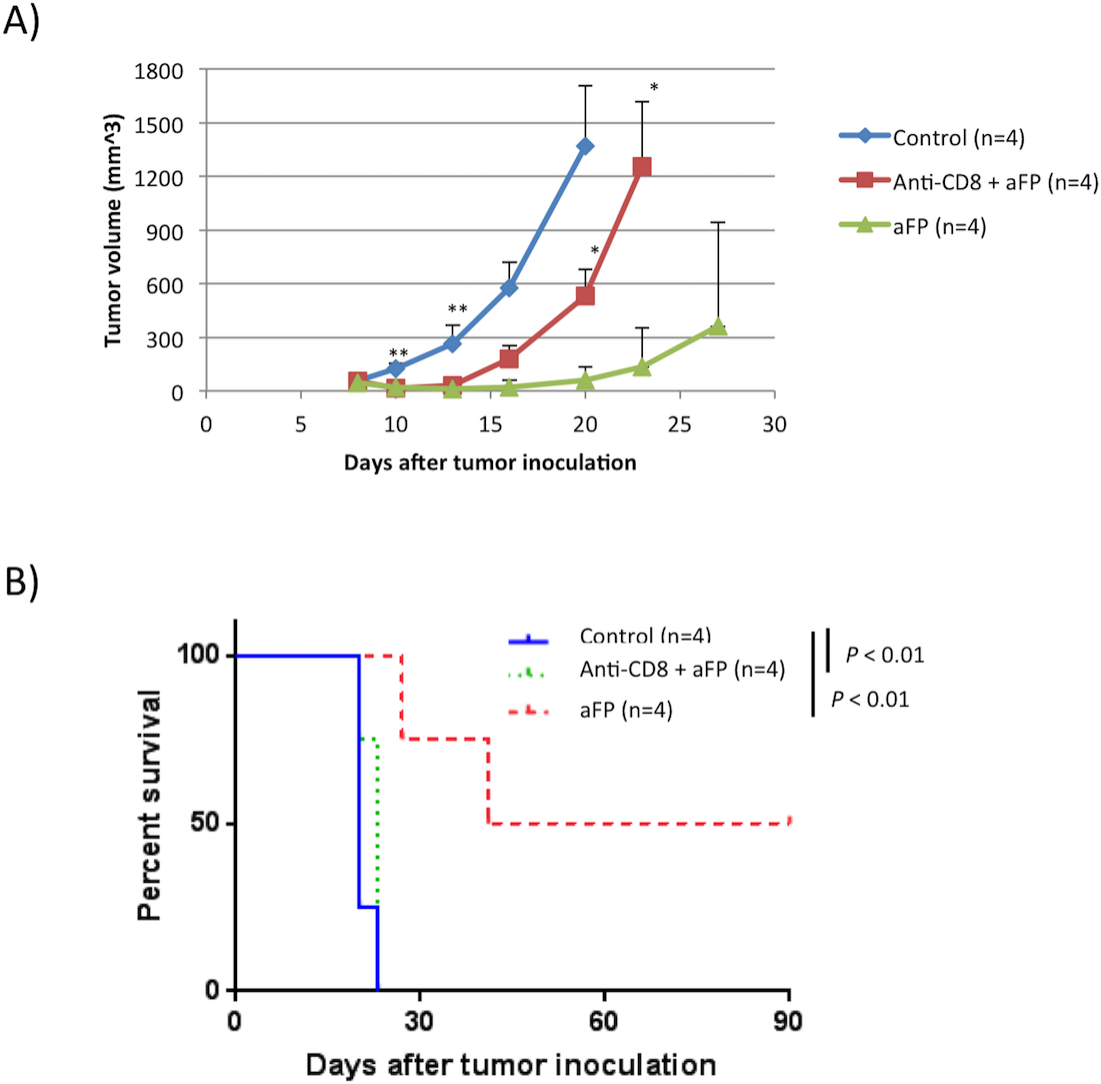
aFP treatment in absence of CD8^+^ T lymphocytes. (A) tumor volume curves of mice in the control group (no treatment), anti-CD8+aFP and aFP group after tumor inoculation. ** *P* < 0.001 comparing control to anti-CD8+aFP or aFP group. * *P* < 0.05 comparing anti-CD8+aFP to aFP group. The bars represent SD. (B) Kaplan-Meier survival curves of mice receiving tumor inoculation. The significance values for the difference between the survival curves are: control or anti-CD8+aFP vs. FP group (*p* < 0.01).

To corroborate our results indicating that the adaptive immune system is necessary to eradicate cancer cells after aFP, we repeated the experiment with anti-CD8 depletion antibody to ablate CD8^+^ T lymphocytes in the mouse. Control group had no treatment. As shown in Fig 8, treatment of aFP in absence of CD8^+^ T lymphocytes failed to prevent tumor growth and eventual euthanasia, while 2 out of 4 mice in aFP group, no CD8^+^ T lymphocytes depletion, survived. (P<0.01; Fig 8B). However even CD8^+^ T lymphocytes was depleted, aFP led to a reduction in tumor volume on day 10 and 12 (P<0.001; Fig 8A). This observation indicates aFP induced apoptosis of cancer cell and then led to a temporal tumor reduction. However that was insufficient to completely remove the tumor because of lack of activated adaptive immunity.

Repeated experiments are shown in S9 Fig.

## Discussion

This is the first study known to the authors that demonstrates marked tumor size reduction and initiation of a systemic, tumor specific immune response using FP laser irradiation in the absence of any additional drugs or adjuvant therapy. FP has been used for a range of clinical indications, however the ability of FP to induce tumor regression is somehow surprising as FP treatments are typically used to stimulate wound healing and tissue tissue regeneration. Further more the use of FP has generally been limited to achieving localized tissue effects within the treatment area. FP is typically used for treatment of non-malignant skin conditions, with few exceptions such as for the treatment of actinic keratoses [11]. To date, the mechanism of overall reduction of actinic keratoses within the treatment field is not known and warrants further investigation.

aFP has been used successfully for treatment of skin cancer in conjunction with PDT treatment [9, 10]. In such treatments, aFP is used merely as a modality to provide a more effective response to the PDT treatment by aFP-assisted drug delivery of the photosensitizer, and not for directly affecting the tumor tissue itself. In some studies, aFP has been used for laser-assisted delivery of a vaccine [24, 25]. The principal role of aFP in vaccine delivery is to improve permeability of the skin barrier and/or to provide a needle-less alternative to classical injection techniques. Nevertheless, some studies have also found that aFP in combination with vaccines may provide a more potent vaccine immune response as compared to mechanical transdermal vaccine delivery [24, 25].

The concept of using aFP for direct irradiation and intentionally only partial ablation of a potentially lethal, metastasizing tumor is novel and in marked contrast to conventional energy-based oncological treatments. The current paradigm of energy-assisted cancer treatment is to cause complete destruction and eradication of the tumor tissue as a direct result of the laser irradiation. The concept of intentionally damaging only a small fraction of the tumor is distinct from (and contrary to) current energy-based approaches for tumor treatment.

Hyperthermia of tumor tissue, including laser-assisted hyperthermia or thermal ablation, has been used previously to induce a systemic tumor response [26]. However, such approaches appear to be directed only to treatment of either the entire tumor tissue or at least a substantial portion thereof. Also, non-fractional laser treatments such as, e.g., pulsed dye laser or Nd:YAG laser treatments have been used to treat local skin cancers [27–29]; however, the objective of these treatments was to completely eradicate a tumor either by direct thermal destruction or by closure of the vessels supplying blood to the tumor, therefore “starving” the tumor cells to death. It is a novel concept and contradicts previous approaches of tumor treatment to intentionally limit the laser induced tissue damage to only to a very small fraction of the tumor volume.

We have demonstrated (without introduction of other bioactive substances) that aFP laser treatment of a particular tumor type can achieve a reduction of tumor size and reject tumor rechallenge. This is indicative of an immune response that bears some similarities to the immune response achieved with PDT treatment [17]. This is a remarkable result, as PDT is dosed and designed to destroy the complete tumor volume, whereas aFP directly destroys only a small percentage of the tumor tissue. The observed tumor reduction and immune response in the present study occurred in the absence of any systemically-delivered photosensitizer or drug.

In this initial study, no assessment of the effects of variation of dosimetry for the tumor treatment was made. The present aFP studies were each performed using a constant energy of 100 mJ per MTZ (e.g., a single 100 mJ pulse was used to form each ablated hole in the aFP irradiation pattern). This energy was chosen as being sufficient to ablate tissue to a substantial depth within the tumor. For example, the MTZs formed by the 100 mJ pulses extended to approximately 2/3 of the tumor depth. This energy per MTZ is high compared to settings routinely used for the most common fractional ablative CO2 laser indications, such as skin rejuvenation and wrinkle reduction, which typically vary from about 15 mJ to 100 mJ depending on the associated pattern density and clinical indication [30]. The dermal thickness of mouse skin is about 0.29 to 0.41 mm [31], which is significantly thinner than the approximately 0.6 to 2.4 mm dermal thickness of human skin [32].

For this study we have selected a density of 5% which could still be applied safely in the absence of risk for bulk heating for such relatively high MTZ energies. We could demonstrate that a thermal injury within a very small percentage of the tumor tissue induced tumor regression and an immune response stimulation.

It should be noted that the morphology and geometry of ablative fractional lesions within colon cancer tumor tissue differ substantially as compared to ablative fractional lesions within skin. In cutaneous tissue, aFP CO2 lesions typically present as regular, straight columns of a laser-induced hole surrounded by a cuff of thermal injury. These laser-generated passages appear to be straight and stable, as they are embedded within a collagen rich-tissue and surrounded by a cuff of coagulated collagen, both of which can provide some stability [3]. In contrast to aFP skin MTZs, the lumen of the laser-created holes in tumor tissue appear to be collapsed; the amount of thermal injury surrounding ablated MTZs in such tumors is minimal, and appears to be less than 40 μm thick as assessed by NBTC staining.

This geometry of aFP lesions in colon cancer can be explained by considering anatomical differences of tumor tissue and skin tissue. First, as the colon cancer tissue grows rapidly, causing volume expansion, there is an increased intratumoral tissue pressure. Although we have not measured the pressure within a tumor, this pressure phenomenon during tumor growth has been observed and described previously [33]. Also, the reduced amounts of collagen within the tumor is expected to result in decreased mechanical stability as compared to skin tissue. It seems plausible that the combination of increased intratumoral pressure and reduced tissue stability can lead to the substantially complete collapse of the laser-ablated holes. Further investigations of the geometry and collapse of MTZs in tumor tissue during aFP procedures may reveal important correlations with tumor-specific biology.

The histology that was performed immediately following aFP revealed an increased amount of erythrocytes in vessels within the tumor volume, suggesting an enhancement of blood flow within the tumor resulting from the aFP. This is another observation that bears further detailed investigation in aFP of tumors. The apparent increase in blood flow in the tumor may facilitate some limited transport of tumor cells out of the tumor, but also may facilitate access of immune competent cells to the core region of the tumor. For example, the enhanced tissue pressure within the core of rapidly-growing tumors can make the core region inaccessible to immune competent cells, which rely on vascular perfusion of the tumor. We speculate that a temporary increase in blood flow may result from the partial but sudden vaporization of tumor tissue and the resulting internal pressure relief during aFP of a tumor. This may facilitate access of immune competent cells to cancer cells within the tumor.

NBTC staining performed immediately after aFP laser exposure revealed that there was an absence of bulk heating in the fractionally-treated tissue volume, indicating a lack of confluent thermal injury within the tumor tissue. This particular thermal injury pattern within the tumor tissue distinguishes aFP of tumor tissue from prior energy-based tumor treatment approaches using physical modalities, such as ionizing radiation therapy or classical thermal ablation approaches, that typically provide a relatively homogenous dose of energy throughout the tumor tissue.

While extensive and homogenous irradiation of tumors may be desirable for tumor destruction, such “full-irradiation” approaches have potential downsides. For example, the substantially complete destruction of the tumor tissue also destroys nearby immune competent cells that might be helpful to trigger an immune response. This is of particular concern, e.g., in radiation therapy because immune competent cells have a low damage threshold and might be even more vulnerable to a full-irradiation treatment than the tumor cells themselves [34]. Current treatments are designed to destroy the tumor, but not to necessarily trigger an immune response. The death pathway varies with different thermal doses, and it is not clear which pathway might be the most effective for stimulating an immune response [35].

A fundamental advantage of the thermal damage pattern unique to FP is that throughout the tumor, cancer cells are exposed to wide range of temperatures that can vary from the normal body temperature of the host up to the vaporization temperatures generated in the MTZs, which may be in excess of 100°C. This wide temperature variation results from the very steep temperature gradient caused by the focused laser radiation within the location of each MTZ vs. the immediately adjacent unexposed tissue. Although only one specific aFP treatment pattern and pulse energy was utilized in the present study, triggering of a marked systemic immune response was observed despite the minimal amount of overall thermal damage done to the tumor volume. We estimate that only approximately 5% of the total tumor volume was thermally damaged.

Neutrophilic infiltrates in tumor tissue have been well described previously and may have positive or negative impact on the immune response. Tumor-associated neutrophils (TANs) are classified in tumor-bearing mice as N1 or N2 [36]. N1 acts as antitumorigenic immunity but N2 acts as protumorigenic immunity such as supporter for tumor growth and suppressor against anti-tumor immune response, depending on the tumor microenvironment [37]. Jablonska et al reported interferon-beta was polarized TAN toward N1 phenotype [38]. Colombo et al reported G-CSF at the tumor site led the neutrophils activated and aquired cytotoxicity [39]. Fridlender et al reported N2 phenotype is deriven by the TGF-beta [36]. N2 phenotype is related with an unfavorable prognosis [40–43]. These reports suggest controlling tumor microenvironment and a neutrophilic shift to N1 phenotype is desirable for to trigger a favorable immuneresponse. There are reports that tumors secrete TGF-beta to evade anti-tumor immunity [44, 45]. The secretion induces a N2 phenotype. Mishalian et al reported in established tumor TAN acuired a more-tumorigenic phenotype [46]. This might be related with increasing secretion of TGF-beta in growing tumor. In our study, aFP induced a Treg level decrease. We hypothesize the following meachanism: (1) necrotic and apoptotic tumor cell caused by thermal injury of aFP lead to tumor reduction and release of damage-associated molecular patterns (DAMPs) [47]. DAMPs recruits neutrophils into the tumor [48] and lead the recruited neutrophil to shift to N1 phenotype [23]. (2) Decreasing TGF-beta secretion prevents the neutrophils to shift to N2 phenotype and Treg to be recruited into the tumor, as TGF-beta is known to recruit Treg [49]. Chaput el al have demonstrated increased CD8^+^ T lymphocyte activity as a result of Treg depletion [50]. Our study also showed Treg suppressed CD8^+^ T lymphocyte activity. This relation between CD8^+^ T lymphocyte activity and Treg makes it plausible that depletion of Treg by aFP led to activate CD8^+^ T lymphocyte in our study. We were able to demonstrate prevent tumor growth after inoculation. Interestingly, inoculated tumor on anti-CD8 antibody-treated mice experience a temporary reduction in tumor size and significant growth delay shortly after a single aFP treatment. We hypothesize neutrophils played a role to reduce the tumor because the reduction cannot be explained by tumor ablation due to direct thermal injury as the volume of the directly thermally damaged tissue was minimal as compared to the reduction in tumor size and B lymphocytes, NK cells and macrophage cells were not increased comparing with control group. In spite of the marked temporary reduction of tumor volume and tumor re-growth no regression was observed in the anti-CD8 antibody-receiving mice. This observation strongly indicates that adaptive immunity is necessary to eradicate cancer cells.

The cancer cell we used in this study is the colon CT26.CL25 cancer cells transduced with lacZ gene to stably express a tumor antigen (beta-gal) which are syngeneic to balb/c, to investigate the effectiveness of aFP treatment against tumor antigens. The cancer cells allowed us to design study models similar in the clinical situation in order to investigate the importance of the expression of the tumor antigen after aFP laser treatment. The cytotoxic lymphocyte cell can recognize and bind the immunodominant peptide epitope derived from beta-gal antigen on MHC class I haplo type H2L^d^ on CT26.CL25. However, the beta-gal expression on the cell in a normal state is not sufficient to stimulate an immune response to prevent tumor growth [17]. There are many reported studies that utilize this tumor-associated antigen (TAA) property to show anti-tumor activity of cancer therapies. Therefore we used aFP as a new approach for releasing TAA and enhancing anti-tumor immunity in this study. Mroz et al showed that PDT induced local remission of CT26.CL25 tumor as well as a systemic tumor-specific immune response, resulting in regression of a remote, untreated antigen-positive tumor, and suggesting release of TAA (beta-gal) from the tumor by PDT induces systemic tumor-specific immune response [17]. In our study, pentamer staining revealed beta-gal epitope specific CD8^+^ T lymphocytes in treated mice, and the cytotoxic assay and rechallenge examination also showed systemic tumor-specific immune response of CD8^+^ T lymphocytes. These results indicate aFP has the potential to induce a systemic tumor-specific immune response. Interestingly the inoculation as rechallenge of CT26WT tumor which was parental tumor of CT26.CL25 was rejected in mice cured from CT26.CL25 tumor. We expect release of common TAA by aFP treatment affects to the rejection. Further investigation of release of the TAA is necessary. There is concern the tumor destruction promotes development of metastasis. However we expect the metastatic cells are killed by the epitope specific CD8 lymphocytes from this study. We observed also one mouse in the control group which had tumor regression. It is feasible that this mouse spontaneously acquired immunity towards the tumor. However most of the mice in the control group did not acquire sufficient enough immune response to survive in contrast to the FP treated group. Mroz et al reported P1A antigen-positive tumor-bearing untreated DBA/2 mice showed slower growth of the tumor and showed better survival than nude mice [51]. This indicates untreated mice also had an induced immune response against TAA by the tumor. There is a report regarding enhancement of the incident of metastasis in surgically tumor-resected mice caused by operative stress [52]. However in our study there was no incidence of metastasis after aFP treatment. This suggests that aFP treatment has only minimal risk for metastasis.

In conclusion, we used aFP to induce tumor regression and systemic tumor immune response in a balb/c mice model bearing CT26.CL25 tumors. Despite the small degree of thermal injury to the overall tumor, and absence of any introduced drug or other bioactive substance, aFP was observed to induce innate, adaptive immunity and prevent tumor recurrence of CT26.CL25 tumors. Moreover, aFP tumor treatment induced long-term immune memory and resistance to rechallenge. These effects might be mediated by tumor antigen-specific cytotoxic T lymphocyte which can recognize the immunodominant epitope of beta-gal induced by aFP irradiation. Accordingly, a marked tumor-specific systemic immune response was produced using limited thermal injury and without administration of any bioactive substances. The effects of aFP at various dosimetry of laser setting, different tumor models, and the effects of combination therapies of aFP with drugs that induce or enhance tumor immunity and their mechanism, will also be investigated in further studies. Although we have chosen a fractional laser modality, other physical modalities capable of inducing a fractional thermal damage pattern, such as focused ultrasound pattern or radiofrequency needle arrays, may also provide some anti-tumor effects and should be investigated.

## Conclusion

The present study indicates that it may be advantageous to apply aFP to tumor tissue to induce an adaptive immune response. It is notable, that such spatially-disperse pattern of thermal damage is distinct from conventional approaches of inducing complete and immediate tumor ablation resulting from a substantially homogenous energy exposure of the entire tumor. We would like to emphasize that aFP treatment of any cancer or malignant lesion (with the exception of actinic keratoses) is at this point considered as investigational, and should not be used as standard of medical care. The results of this animal study encourage further studies to carefully investigate the highly promising role of aFP treatments in oncology.

## Materials and methods

### Cell lines

CT26.CL25 murine colon cancer cell lines (ATCC, Mannassas, VA) was cultured in Dulbecco’s Modified Eagle’s Medium supplemented with 10% heat-inactivated fetal bovine serum, penicillin (100 U/mL) and streptomycin (100 mg/mL) (all from Sigma-Aldrich, Natick, MA) at 37°C in 5% CO_2_. Culturing was performed in 75 cm^2^ flasks (Falcon, Invitrogen, Carlsbad, CA). CT26.CL25 cells were cultured in a constant presence of 500 μg/mL G418 antibiotic (Sigma-Aldrich, Natick, MA) in order to maintain a constant expression of the vector

### Animals

Six-week-old female BALB/c mice (Charles River Laboratories, Boston, MA) were used for the study. The care and handling of the animals were done in accordance with a protocol approved by the Subcommittee on Research Animal Care (IACUC) at Massachusetts General Hospital (MGH).

### Animal tumor model

Mice were inoculated unilaterally at the left leg with 3.5 x 10^5 CT26.CL25 intradermally into the depilated thigh after being anesthetized through intraperitoneal injection of a cocktail of ketamine (90 mg/kg) and xylazine (10mg/kg). Anti-CD8 depletion antibodies (2.43; BioXCell,West Lebanon, NH) were administered intraperitoneally at a dose of 200 ug per mouse every 3 days from one day before tumor inoculation to removal of mice as endpoint. The tumor volume was determined by measuring the longest dimension and orthogonal dimension of the tumor with vernier calipers. Tumor volumes were calculated according to the formula volume = 4π/3×[(a+b)/4]^3^, where a and b represent the long and short axis lengths, respectively. If mice appear to be pain as judged by behavioral traits (flinching, guarding, ruffled fur, pronounced inactivity) during the course of the experiment, they received buprenorphine (0.05 mg/kg) injected subcutaniously or IP every 12 hours for 1 day. If this fails to relieve their pain they were withdrawn from the study and euthanized by transcardial perfusion using 4% paraformaldehyde or zinc fixative (BD, Bioscience, Franklin Lakes, NJ) after mice are anesthetized by intraperitoneal injection of a cocktail of ketamine (180 mg/kg) and xylazine (20mg/kg).Other indications for removal of mice from the study include tumor size exceeding 1.5 cm in length, signs of systemic illness and life threatening burn injury manifested by pronounced inactivity, torpor, ruffled fur, failure to eat and drink and weight loss of 15% or more. If the tumor cell line injection results in ulcer or laser treatment causes unexpected burn or ulcer the mouse was euthanized.

### Fractional CO_2_ laser irradiation

Ablative fractional laser exposure was performed on the tumor site at day 8 after tumor cell inoculation. At that time, the tumors have reached a typical diameter of about 5 mm. Exposures were performed with an Ultrapulse Encore CO_2_ laser (Lumenis Inc, Yokneam, Israel). A single aFP treatment was performed within a treatment area of 6×6 mm, with a pulse energy of 100 mJ energy per pulse, at a nominal density of 5% (i.e., 5% within the treatment area surface was irradiated), one pass, and a repetition frequency of 300 Hz between adjacent MTZs. No skin cooling was applied and the anesthesia was performed by intraperitoneal injection of a cocktail of ketamine (90 mg/kg) and xylazine (10mg/kg).

### Histological tissue processing

The extent of thermal injury was determined immediately after laser exposure of the tumor with nitro-blue tetrazolium chloride (NBTC) staining of frozen sections [53]. Tissue samples were embedded in OCT compound (Sakura Finetek USA, Inc., Torrance, CA), frozen, and sliced vertically and horizontally into serial 20-μm-thick sections in a cryostat (QS11, Avantik Biogroup, Springfield, NJ). Frozen sections were incubated with NBTC solution for 4 hours and counter-stained with eosin. The solution was made with 80 mg of nitro blue tetrazolium dissolved in 200ml of PBS and 100 mg of β-nicotinamide adenine dinucleotide (all from Sigma-Aldrich, Natick, MA). The lesion geometry and histology were assessed by hematoxylin and eosin (H&E)-stained paraffin sections. Tissue fixation was obtained by performing transcardial perfusion using 4% paraformaldehyde or zinc fixative (10 ml/ each mouse) immediately after aFP, the aFP-treated tissue samples were excised, and the aFP-treated tissue samples were placed into formalin. After 72 hours fixation in formalin, each tissue sample was processed for paraffin embedding, sliced vertically and horizontally into serial 5-μm-thick sections, and stained with H&E. The lesion geometry and aFP laser penetration depth were assessed using a standard light microscope with a calibrated scale bar. Representative histologies were photodocumented. The samples which were used to determine the dimensions of the aFP-treated tissue samples were procured immediately after aFP.

### Rechallenge

Mice surviving 90 days after tumor inoculation were rechallenged with matched tumor cells (3.5 x 10^5 CT26.CL25) intradermally in the contralateral (right) thigh against previous inoculation. Age-matched naive mice were inoculated with the same number of the cells in the right thigh as the controls were. The inoculated mice were monitored for another 60 days to confirm tumorigenesis. Additionally Mice surviving 60 days after rechallenge experiment with CT26.CL25 cells were rechallenged with CT26 wild type (CT26WT) colon carcinoma cells (3.5 x 10^5), which is parental cells of CT26.CL25 cells intradermally in the left thigh.

### Immunohistochemistry

To confirm whether innate immunity was induced after the fractional laser treatment, immunohistochemistry was performed. Tumors were excised on day 9 (1 days after aFP) and paraffin sections were made using zinc fixative (BD, Bioscience, Franklin Lakes, NJ) in the same manner as mentioned above. Slides were deparaffinized and washed with PBS or 0.1% triton solution 5 minutes 3 times. Slides were incubated with 10% goat serum in PBS solution for 60 minutes at room temperature (RT) and next a 1:200 dilution of rat monoclonal anti-Ly-6G antibody (Abcam, Cambridge, MA) or anti-CD206 antibody (Abcam), or a 1:100 dilution of rabbit monoclonal anti-cleaved caspase 3 antibody (Cell Signaling Technology, Danvers, MA) in 10% goat serum in PBS was added for overnight incubation at 4°C. A 1:200 dilution of fluorescence-labeled secondary goat anti-rat IgG antibody (Life technology, Carlsbad, CA) in 10% goat serum in PBS was added for 60 minutes at RT on next day. DAPI (Life technology) was used as a nuclear counterstain. To observe fluorescence, a confocal microscope with wave lengths of 405, 561 and 640 nm was used (Olympus, Tokyo, Japan).

### Flow cytometry analysis

To confirm regulatory T cell (Treg), CD8 lymphocyte and neutrophil were affected by aFP, flow cytometry analysis was performed. For leukocyte isolation from tumor, fresh CT26.CL25. tumors were dissociated mechanically filtering through 70 μm strainer on 6 well culture plate with DNaseI (10 μg/ml, Roche; Nutley, NJ) and collagenase (10 mg/ml, Life technologies) and incubated 60 minutes at 37°C. The dissociated cells were positively selected for CD45 surface expression using CD45 MicroBeads and magnetic-activated cell sorting (MACS) system (Miltenyi Biotec Inc., Auburn, CA). Then the cells were stained with antibodies CD3-FITC (145-2C11; eBioscience; Santa Clare, CA), CD4-APC-eFluor780 (RM4-5; eBioscience), CD8a-PE/CY7 (53–6.7; eBioscience), CD11b-PE (M1/70; eBioscience), CD19-APC (eBio1D3; eBioscience), CD25-PerCP/Cy5.5 (PC61.5; eBioscience), NKp46-PE (PE 29A1.4; eBioscience), F4/80-PE (BM8; eBioscience) or Ly-6G-PerCP/Cy5.5 (1A8; Bioledgend), for 30 minutes at 4°C after they were blocked at 4°C for 15 minutes using PBS containing 5% BSA and 0.1% sodium azide. Following staining for surface markers, cells were fixed and permeabilized by Foxp3 / Transcription Factor Staining Buffer Set (eBioscience) according to the manufacturer’s instructions at room temperature for 30 minutes. After the permeabilization the cells were stained with Foxp3-APC antibody (MF-14; Bioledgend) at 4°C over night. Next day the stained cells were analyzed on FACSCanto (BD Bioscience, Franklin Lakes, NJ).

### Pentamer staining

To confirm epitope specific CD8^+^ T lymphocytes were elicited by aFP treatment, we stained isolated leukocytes from tumor with epitope (TPHPARIGL) specific pentamer (ProImmune, Oxford, UK) costaining with anti CD3-FITC (145-2C11; eBioscience; Santa Clare, CA), CD4-APC-eFluor780 (RM4-5; eBioscience) and CD8a-PE/CY7 (53–6.7; eBioscience). The assay was conducted according to the manufacturer’s instructions.

### Cytotoxicity assay and Treg function experiment

To acquire more evidence for the involvement of adaptive immunity, we measured CD8^+^ T lymphocytes -cytotoxicity against CT26.CL25 colon carcinoma cells using CD8^+^ T lymphocytes sorted from TILs with/without CD25 (which is one of the marker of the Treg) positive CD4^+^ T lymphocytes sorted from the drainage lymph node. Cytotoxicity was assessed by Galacto-Light Plus System (Thermo Fisher Scientific). 5 days after aFP, leukocytes isolation from tumor and magnetically positive selection by MACS described above and lymphocytes isolation from tumor drainage lymph node using 70-mm mesh nylon cell strainer (BD Falcon) were performed. Then CD8^+^ T lymphocytes from TILs and CD4^+^ CD25^+^ T lymphocytes from the lymph node were sorted using FACSAria Fusion Cell Sorter (BD Biosciences), with antibodies CD8a-PE/CY7 (53–6.7; eBioscience), CD4- APC-eFluor780 (RM4-5; eBioscience) and CD25- PerCP/Cy5.5 (PC61.5; eBioscience).7.5x10^2 of sorted CD8^+^ T lymphocytes from tumor of aFP-treated mice and 100 target cells (CT26.CL25) were dispensed with or without 5.0x10^2 CD4^+^ CD25^+^ T lymphocytes from tumor drainage lymph node of untreated tumor-bearing mice (control group) in 150μL of RPMI with 10% heat inactivated fetal bovine serum, penicillin (100 U/mL) and streptomycin (100 mg/mL) (all from Sigma, St Louis, MO) to wells of round bottom 96-well plate. Simultaneously all wells were stimulated with anti-CD3/anti-CD28 antibodies (5 μg/mL and 2 μg/mL respectively; eBioscience) in the presence of IL-2 (20ng/mL; Bioledgend). CD4^+^ CD25^+^ T lymphocytes were stimulated with TGF-beta (5ng/mL; eBioscience) additinally. 24h after incubation at 37°C in 5% CO2, 10μL of supernatant was used to assess beta-galactosidase enzyme activity using Galacto-Light Plus System according to manufacturer’s instructions. The percentage of specific lysis was calculated according to formula: {test beta-galactosidase enzyme release- spontaneous beta-galactosidase enzyme released}/{maximum beta-galactosidase enzyme released–spontaneous beta-galactosidase enzyme released}. The maximal release was obtained by incubation of target cells in lysis solution contained in the kit.

### Statistics

All experiments were repeated at least twice. All statistical analyses were performed with GraphPad Prism 7.0 (GraphPad Software). All values are expressed as the mean ± SD. Flow cytometric results and cytotoxicity assay were compared with a two-tailed Mann-Whitney test. Treg function examination was analyzed with a two-tailed Wilcoxon matched pairs signed rank test. Tumor volumes were compared with a two-tailed Mann-Whitney test (two groups) or one-way ANOVA (three groups). Survival analysis was performed using the Kaplan-Meier method and a log-rank test. Values of P < 0.05 were considered statistically significant.

## Supporting information

S1 Fig Flow cytometric analysis for tumor infiltrating NK cells, B lymphocytes and macrophages. (A) proportion of NK cell, B lymphocytes and macrophage compared with CD45 positive leukocytes in the tumor 2 days after aFP of flow cytometric analysis respectively. There are no significant differences in the cells between both groups. (B) proportion of Foxp3^+^ cells of CD4^+^ CD25^+^ in the TILs 5 days after aFP of flow cytometric analysis. (C) proportion of Foxp3^+^ cells of CD4^+^ CD25^+^ in the tumor drainage lymph node 5 days after aFP of flow cytometric analysis.
(PDF)

S2 Fig Tumor volume and survival curves after aFP treatment in repeated experiment. (A and B) tumor volume curves of mice in the control group and aFP group after tumor inoculation. *** *P* < 0.0005. The bars represent SD. (C and D) tumor volume curve of mice in the control group, and tumor volume curves of cured mice and non-cured mice which are split from original curve in aFP group. * *P* < 0.01, ** *P* < 0.005, *** *P* < 0.0005 comparing control to aFP-non cured group. The bars represent SD. (E and F) Kaplan-Meier survival curves of mice receiving tumor inoculation. The significance values for the difference between the survival curves are: control vs. FP (*p* < 0.0001).
(PDF)

S3 Fig Tumor volume and survival curves after rechallenge test in repeated experiment. (A and B) Kaplan-Meier survival curves of mice receiving the rechallenge test with CT26.CL25 cells. The significance values for the difference between the survival curves are: control mice vs. survival mice (p<0.005). (C and D) Kaplan-Meier survival curves of mice receiving the rechallenge test with CT26 wildtype cells which is parental tumor of CT26.CL25 cells. The significance values for the difference between the survival curves are: control mice vs. survival mice (p<0.005).
(PDF)

S4 Fig Flow cytometric analysis for tumor infiltrating neutrophils in repeated experiment. (A and B) proportion of neutrophil compared with CD45^+^CD3^-^ leukocytes in the tumor on day 1 after aFP treatment.
(PDF)

S5 Fig Immunochemical staining for tumor infiltrating neutrophils in repeated experiment. (A-J) immunohistochemical staining for neutrophil in the tumor 1 days after aFP in the control group and aFP group respectively. Cells stained as red color are neutrophils. Cells stained as yellow color are neutrophils expressing CD206.
(PDF)

S6 Fig Immunochemical staining for apoptotic tumor cells in repeated experiment. (A and B) Immunohistochemical staining for apoptotic cells in the tumor 1 day after aFP in the control group and aFP group respectively. Representative images are shown. Cells stained as red color, which are indicated by white allow head are apoptotic cells.
(PDF)

S7 Fig Flow cytometric analysis for tumor infiltrating lymphocytes in repeated experiment. (A and B) proportion of CD8^+^ T lymphocytes compared with CD3^+^ T lymphocytes in the tumor. (C and D) proportion of Treg compared with CD3^+^ T lymphocytes in the tumor. (E and F) proportion of Treg compared with CD4^+^ T lymphocytes in the tumor. (G and H) proportion of beta-gal epitope specific CD8^+^ T lymphocytes compared with total CD8^+^ T lymphocytes in the tumor. (I and J) proportion of CD8^+^ T lymphocytes compared with Treg in the tumor.
(PDF)

S8 Fig Cytotoxicity assay and Treg function examination in repeated experiment. (A and B) the number in the figures represents % specific lysis of sorted CD8^+^ T lymphocytes from TILs against CT26.CL25 cells. Average specific lysis against CT26.CL25 cells in the aFP group was significantly higher than in the control group (*P* < 0.05). (C and D) percentage of specific lysis CT26.CL25 by sorted CD8 lymphocytes from TILs with and without CD4^+^CD25^+^ T lymphocytes (sorted from tumor drainage lymph node in control group).
(PDF)

S9 Fig aFP treatment in absence of CD8^+^ T lymphocytes in repeated experiment. (A and B) tumor volume curves of mice in the control group (no treatment), anti-CD8+aFP and aFP group after tumor inoculation. * *P* < 0.05 comparing control to anti-CD8+aFP or aFP group. ** *P* < 0.05 comparing anti-CD8+aFP to aFP group. *** *P* < 0.05 comparing aFP group to control or anti-CD8+aFP group. The bars represent SD. (C and D) Kaplan-Meier survival curves of mice receiving tumor inoculation.
(PDF)
