# Supplementary figures and images for: Fractional laser exposure induces neutrophil infiltration (N1 phenotype) into the tumor and stimulates systemic anti-tumor immune response
Source: PLoS One. 2017 Sep 18;12(9):e0184852. doi: 10.1371/journal.pone.0184852 (PMC5602663; doi:10.1371/journal.pone.0184852)

A)

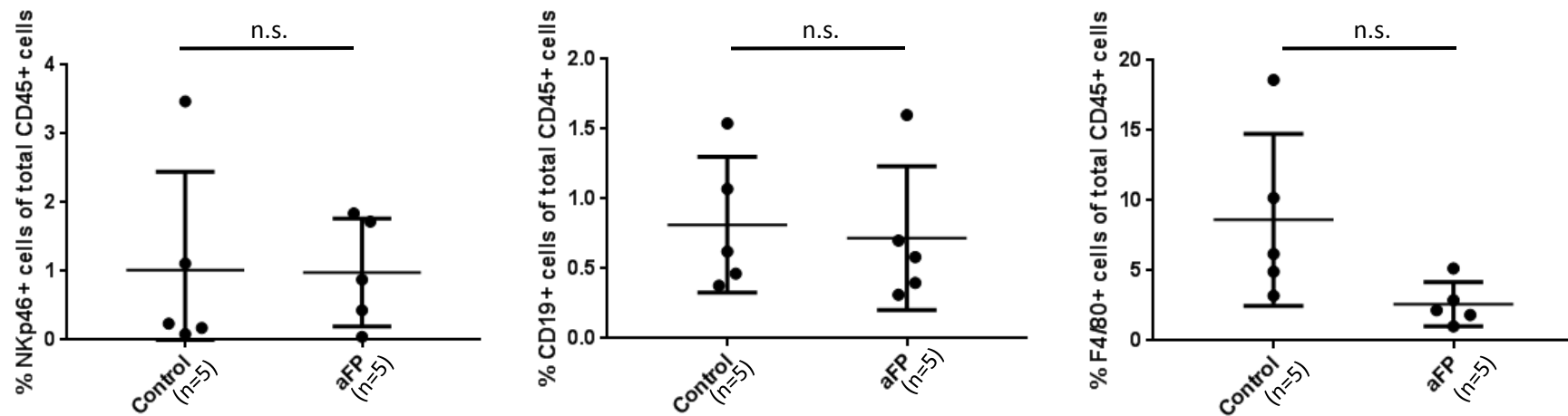

B)

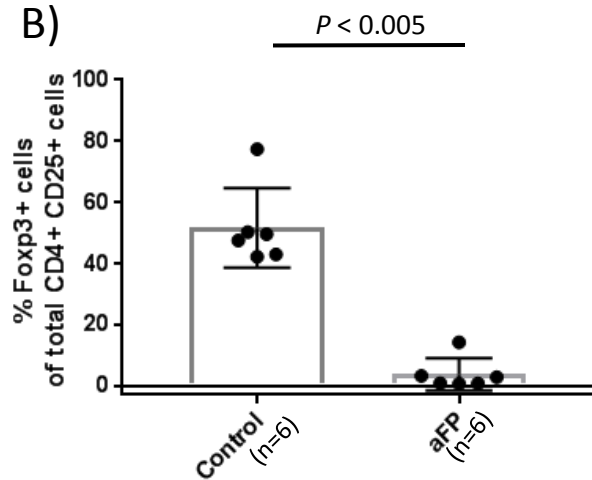

C)

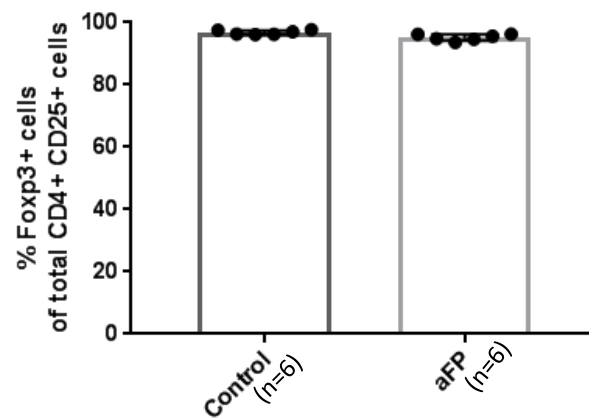

Supplement: S1 Fig — (A) proportion of NK cell, B lymphocytes and macrophage compared with CD45 positive leukocytes in the tumor 2 days after aFP of flow cytometric analysis respectively. There are no significant differences in the cells between both groups. (B) proportion of Foxp3+ cells of CD4+ CD25+ in the TILs 5 days after aFP of flow cytometric analysis. (C) proportion of Foxp3+ cells of CD4+ CD25+ in the tumor drainage lymph node 5 days after aFP of flow cytometric analysis. (PDF) [file pone.0184852.s001.pdf]

Figure S 2

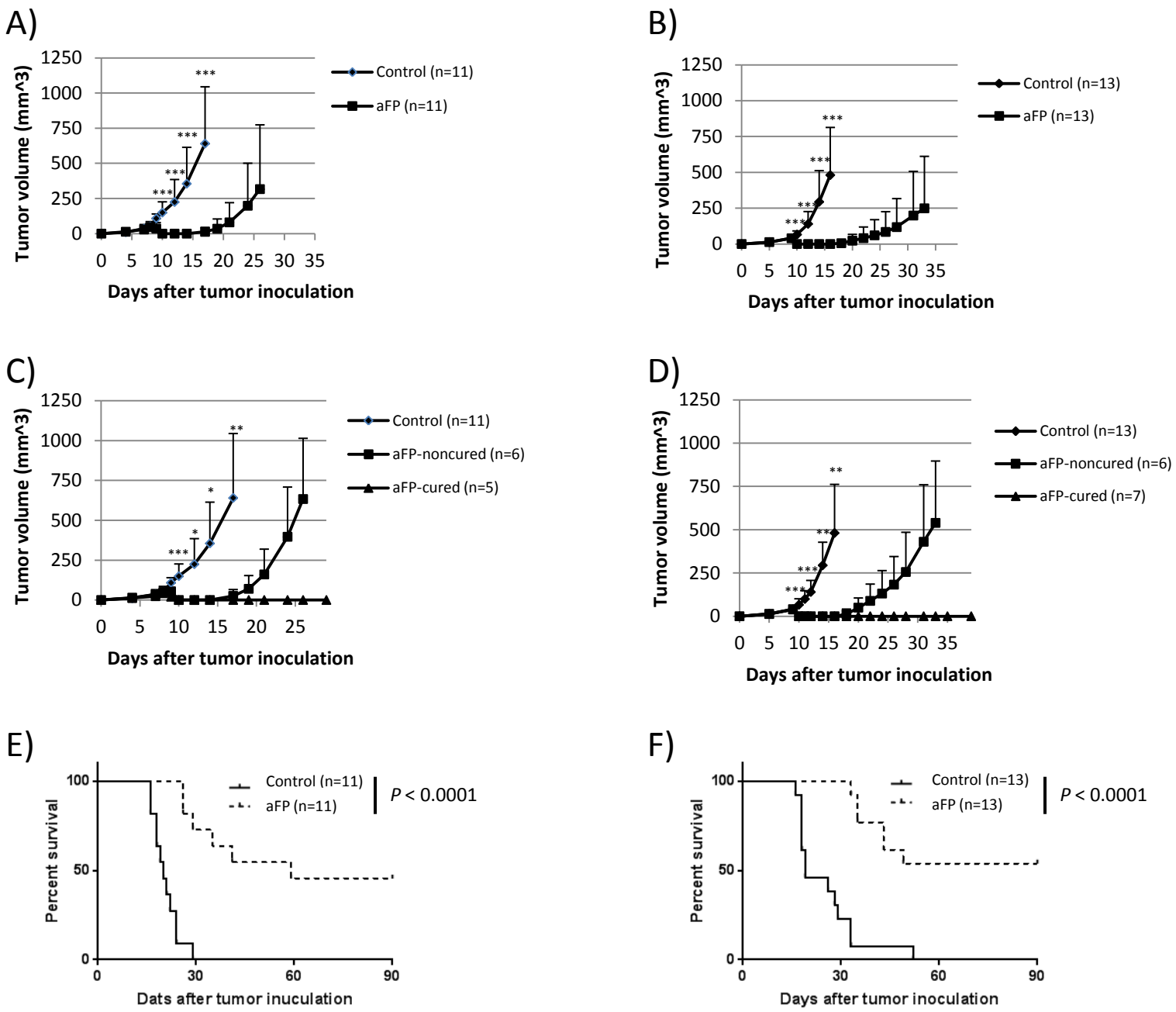

Supplement: S2 Fig — (A and B) tumor volume curves of mice in the control group and aFP group after tumor inoculation. *** P < 0.0005. The bars represent SD. (C and D) tumor volume curve of mice in the control group, and tumor volume curves of cured mice and non-cured mice which are split from original curve in aFP group. * P < 0.01, ** P < 0.005, *** P < 0.0005 comparing control to aFP-non cured group. The bars represent SD. (E and F) Kaplan-Meier survival curves of mice receiving tumor inoculation. The significance values for the difference between the survival curves are: control vs. FP (p < 0.0001). (PDF) [file pone.0184852.s002.pdf]

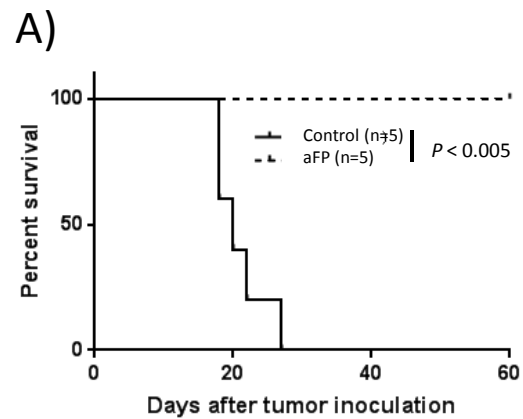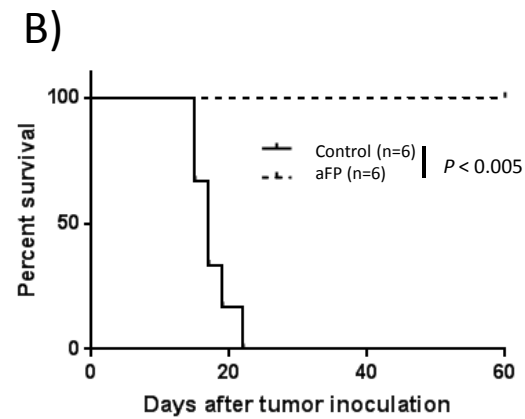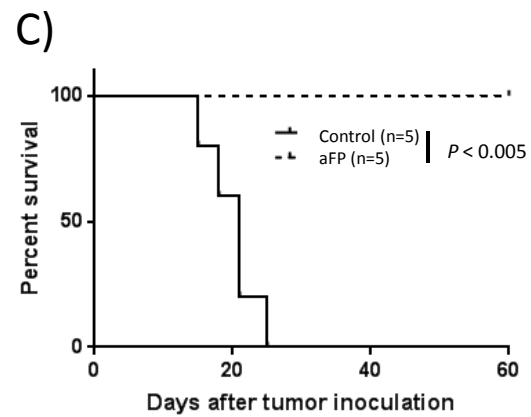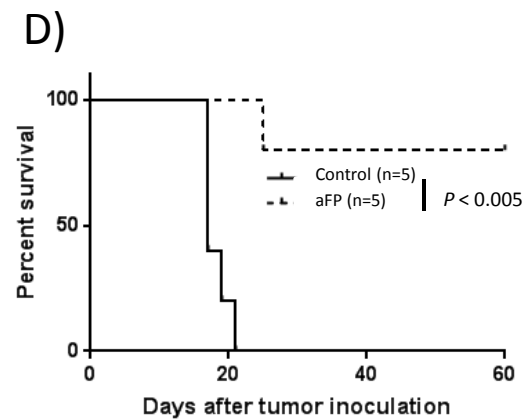

Figure S 3

Supplement: S3 Fig — (A and B) Kaplan-Meier survival curves of mice receiving the rechallenge test with CT26.CL25 cells. The significance values for the difference between the survival curves are: control mice vs. survival mice (p<0.005). (C and D) Kaplan-Meier survival curves of mice receiving the rechallenge test with CT26 wildtype cells which is parental tumor of CT26.CL25 cells. The significance values for the difference between the survival curves are: control mice vs. survival mice (p<0.005). (PDF) [file pone.0184852.s003.pdf]

Figure S 4

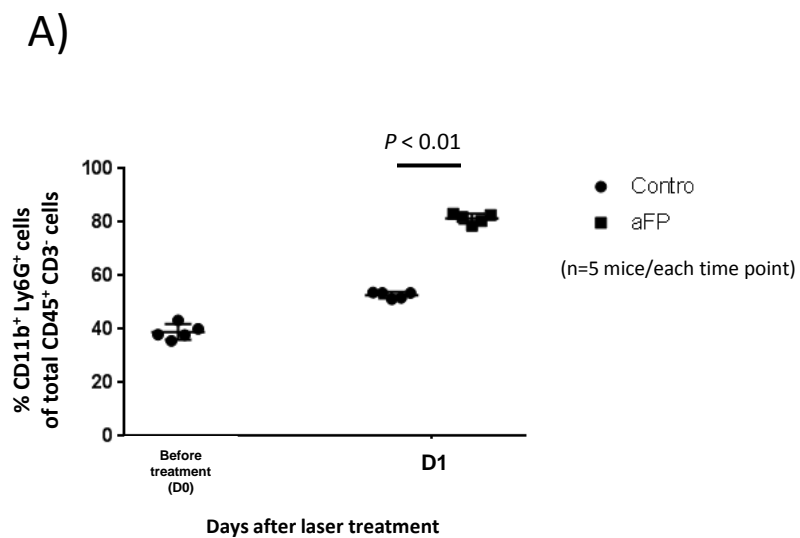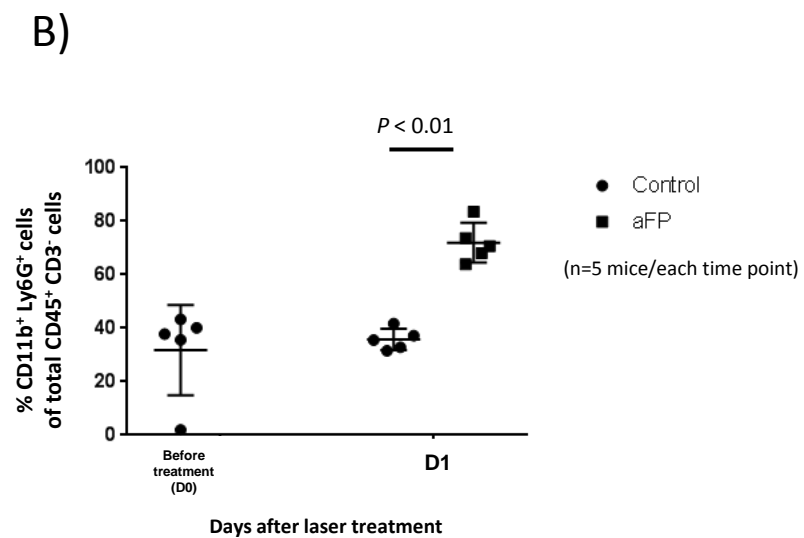

Supplement: S4 Fig — (A and B) proportion of neutrophil compared with CD45+CD3- leukocytes in the tumor on day 1 after aFP treatment. (PDF) [file pone.0184852.s004.pdf]

A) **Control**

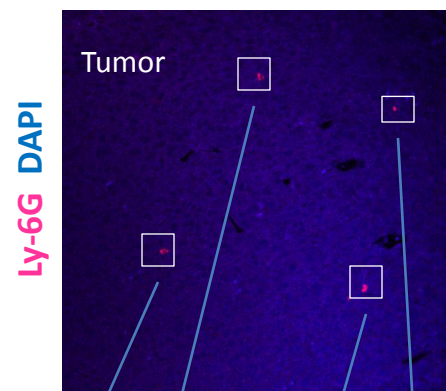

C) **Ly-6G DAPI**

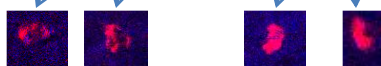

D) **CD206 DAPI**

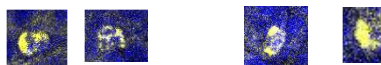

F) **Control**

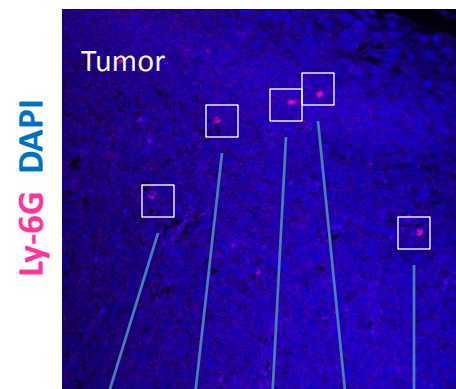

H) **Ly-6G DAPI**

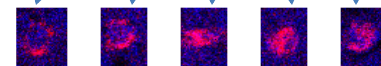

I) **CD206 DAPI**

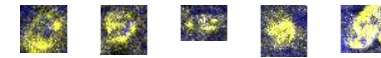

B) **aFP**

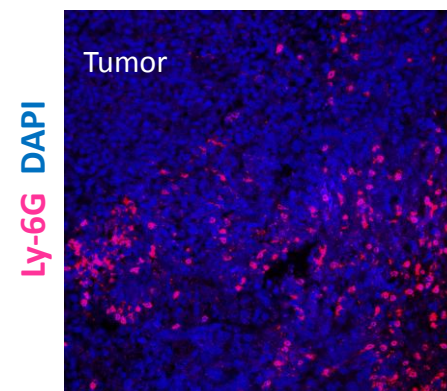

G) **aFP**

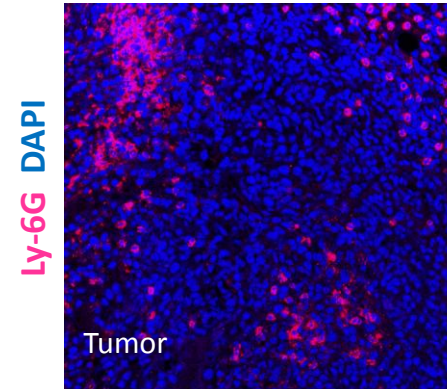

E) **aFP**

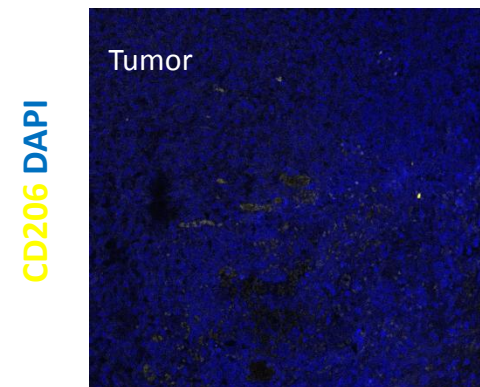

J) **aFP**

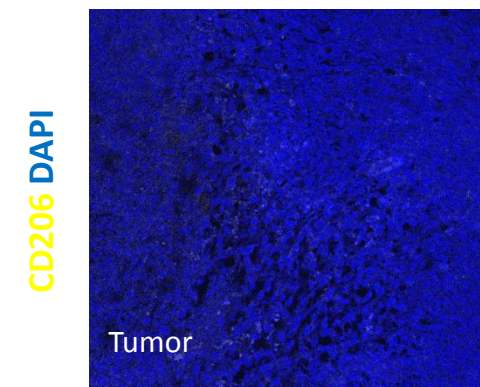

Supplement: S5 Fig — (A-J) immunohistochemical staining for neutrophil in the tumor 1 days after aFP in the control group and aFP group respectively. Cells stained as red color are neutrophils. Cells stained as yellow color are neutrophils expressing CD206. (PDF) [file pone.0184852.s005.pdf]

A)

Control

Cleaved caspase 3 DAPI

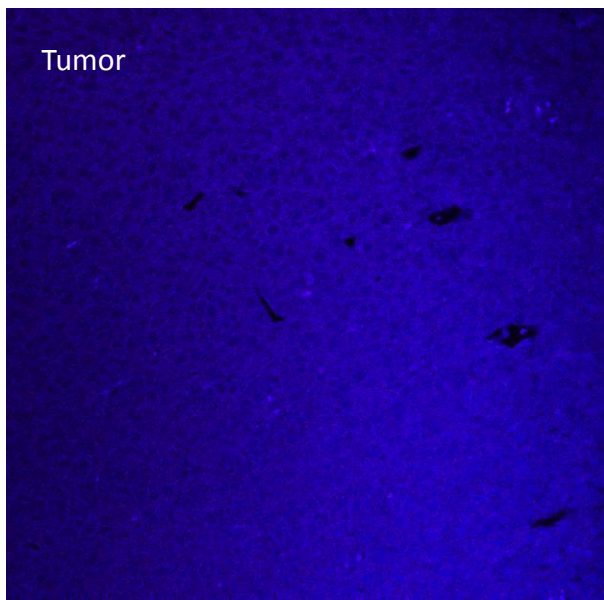

B)

aFP

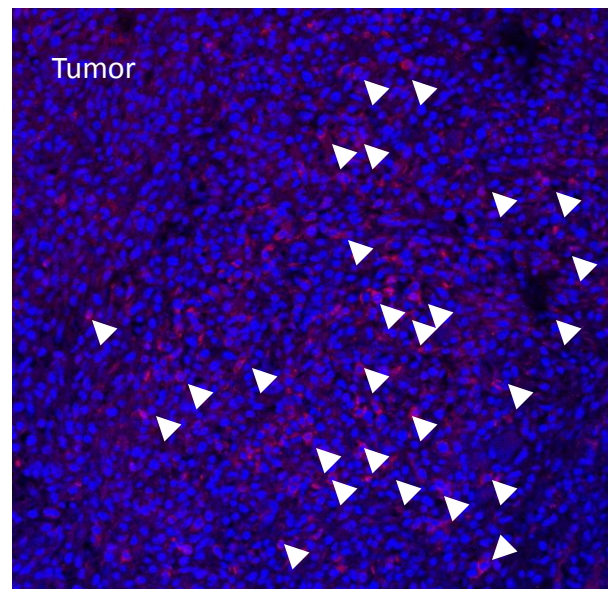

C)

Cleaved caspase 3 DAPI

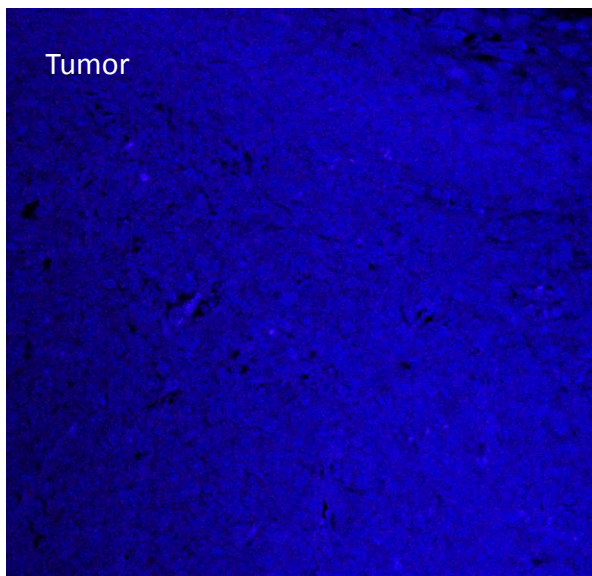

D)

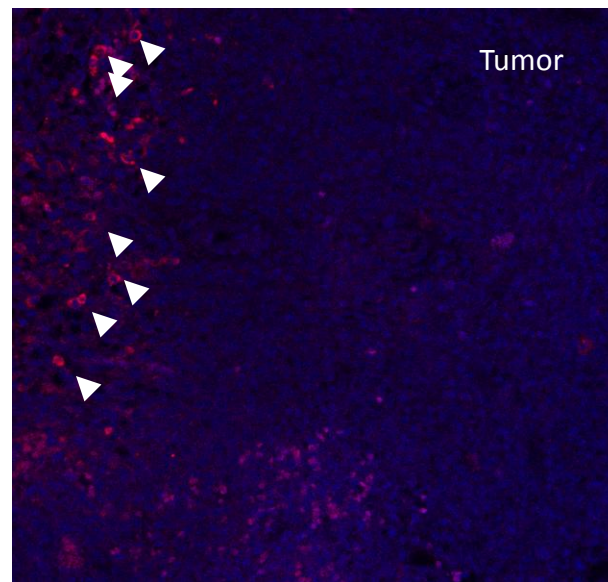

Supplement: S6 Fig — (A and B) Immunohistochemical staining for apoptotic cells in the tumor 1 day after aFP in the control group and aFP group respectively. Representative images are shown. Cells stained as red color, which are indicated by white allow head are apoptotic cells. (PDF) [file pone.0184852.s006.pdf]

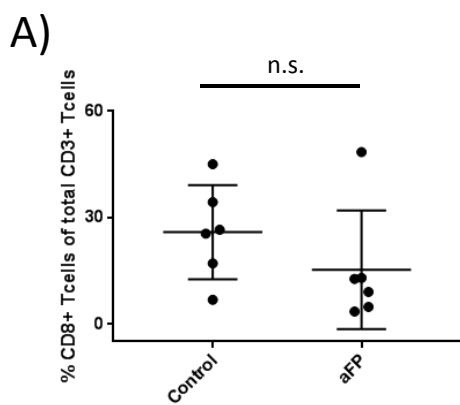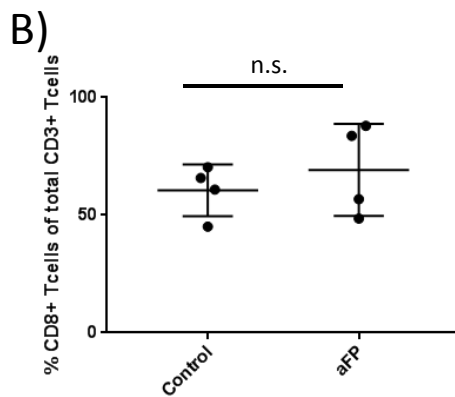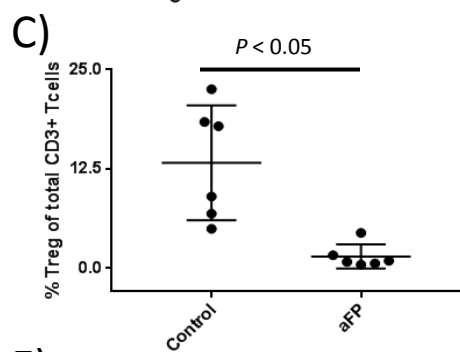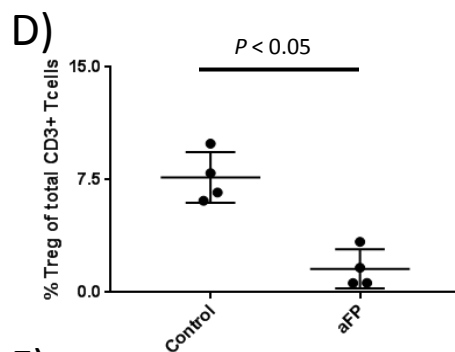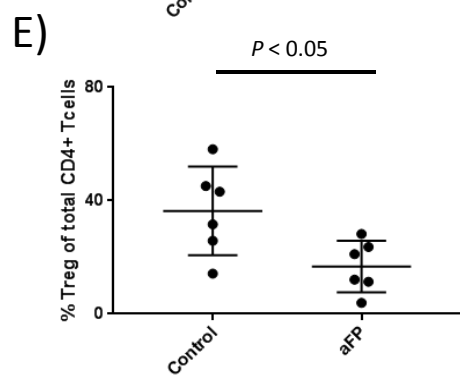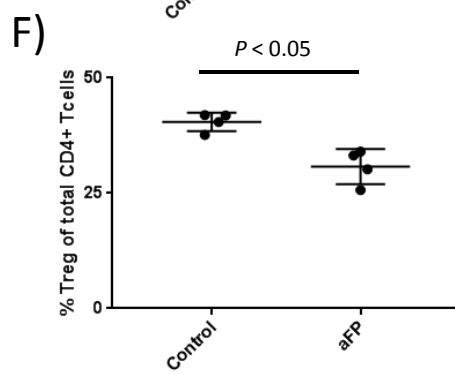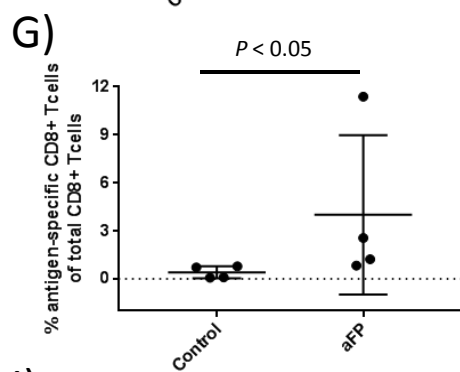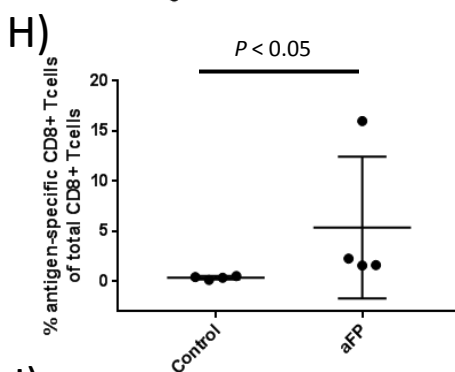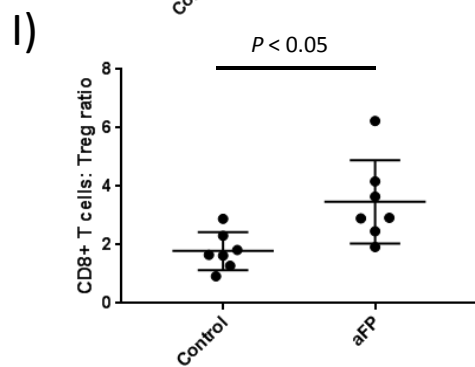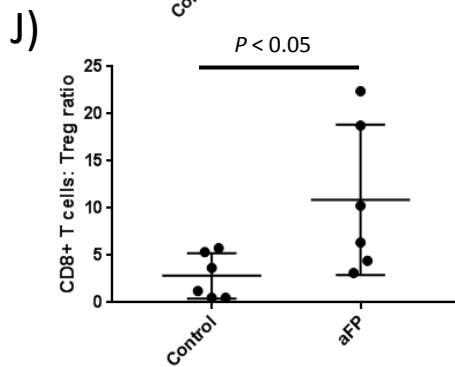

Supplement: S7 Fig — (A and B) proportion of CD8+ T lymphocytes compared with CD3+ T lymphocytes in the tumor. (C and D) proportion of Treg compared with CD3+ T lymphocytes in the tumor. (E and F) proportion of Treg compared with CD4+ T lymphocytes in the tumor. (G and H) proportion of beta-gal epitope specific CD8+ T lymphocytes compared with total CD8+ T lymphocytes in the tumor. (I and J) proportion of CD8+ T lymphocytes compared with Treg in the tumor. (PDF) [file pone.0184852.s007.pdf]

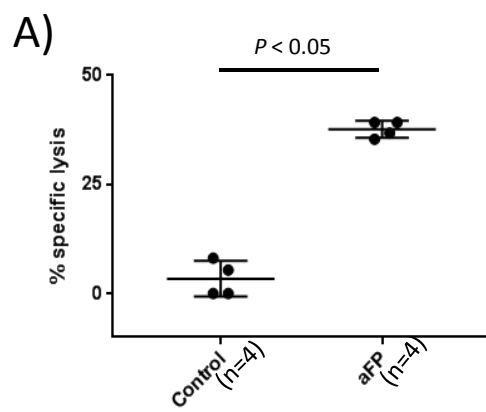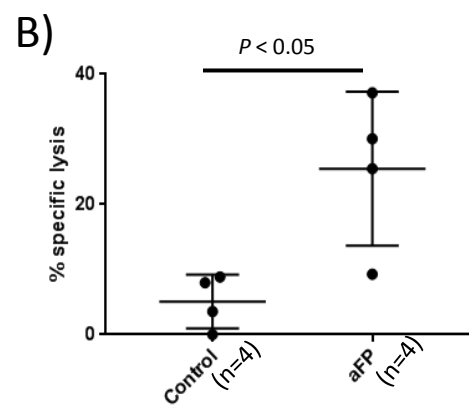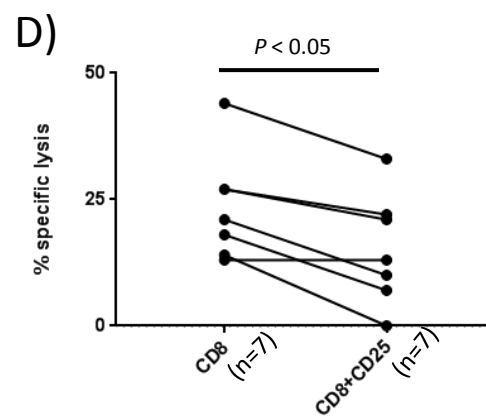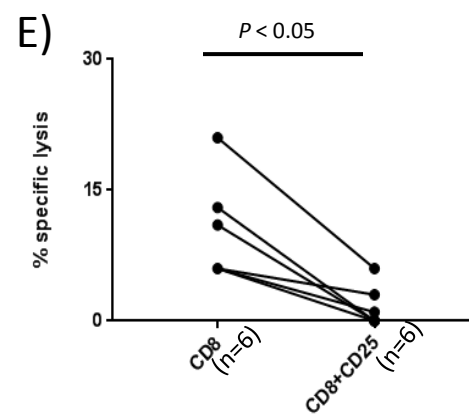

Figure S 8

Supplement: S8 Fig — (A and B) the number in the figures represents % specific lysis of sorted CD8+ T lymphocytes from TILs against CT26.CL25 cells. Average specific lysis against CT26.CL25 cells in the aFP group was significantly higher than in the control group (P < 0.05). (C and D) percentage of specific lysis CT26.CL25 by sorted CD8 lymphocytes from TILs with and without CD4+CD25+ T lymphocytes (sorted from tumor drainage lymph node in control group). (PDF) [file pone.0184852.s008.pdf]

A)

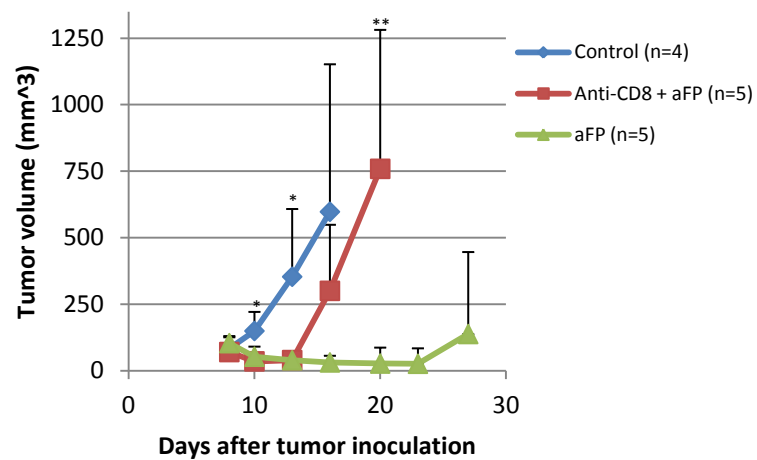

B)

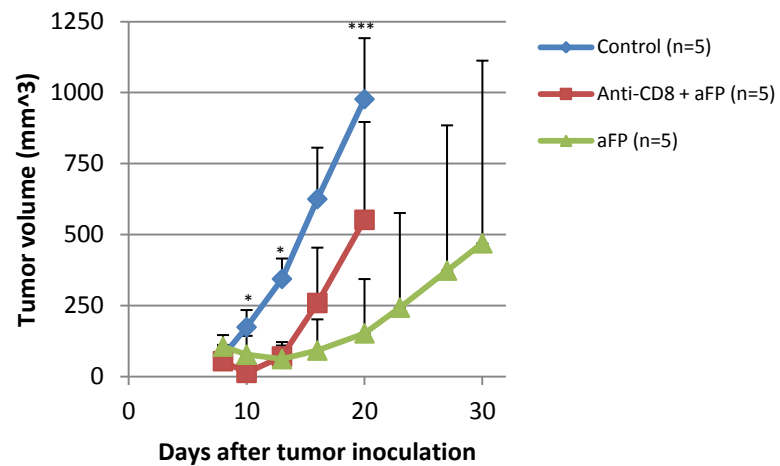

C)

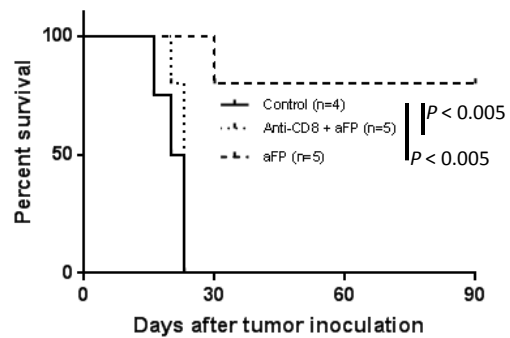

D)

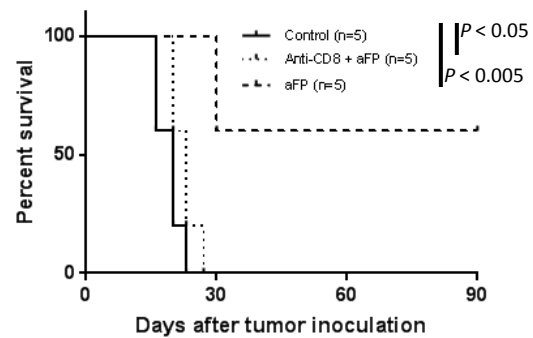

Supplement: S9 Fig — (A and B) tumor volume curves of mice in the control group (no treatment), anti-CD8+aFP and aFP group after tumor inoculation. * P < 0.05 comparing control to anti-CD8+aFP or aFP group. ** P < 0.05 comparing anti-CD8+aFP to aFP group. *** P < 0.05 comparing aFP group to control or anti-CD8+aFP group. The bars represent SD. (C and D) Kaplan-Meier survival curves of mice receiving tumor inoculation. (PDF) [file pone.0184852.s009.pdf]
